# Aqueous proteins help predict the response of patients with neovascular age-related macular degeneration to anti-VEGF therapy

**DOI:** 10.1172/JCI144469

**Published:** 2022-01-18

**Authors:** Xuan Cao, Jaron Castillo Sanchez, Aumreetam Dinabandhu, Chuanyu Guo, Tapan P. Patel, Zhiyong Yang, Ming-Wen Hu, Lijun Chen, Yuefan Wang, Danyal Malik, Kathleen Jee, Yassine J. Daoud, James T. Handa, Hui Zhang, Jiang Qian, Silvia Montaner, Akrit Sodhi

**Affiliations:** 1 Wilmer Eye Institute, Johns Hopkins University School of Medicine, Baltimore, Maryland, USA.; 2 Department of Oncology and Diagnostic Sciences, School of Dentistry and Department of Pathology, School of Medicine, Greenebaum Cancer Center, University of Maryland, Baltimore, Maryland, USA.; 3 Department of Pathology, Johns Hopkins University, Baltimore, Maryland, USA.

**Keywords:** Ophthalmology, Clinical practice, Complement, Expression profiling

## Abstract

**Background:**

To reduce the treatment burden for patients with neovascular age-related macular degeneration (nvAMD), emerging therapies targeting vascular endothelial growth factor (VEGF) are being designed to extend the interval between treatments, thereby minimizing the number of intraocular injections. However, which patients will benefit from longer-acting agents is not clear.

**Methods:**

Eyes with nvAMD (*n =* 122) underwent 3 consecutive monthly injections with currently available anti-VEGF therapies, followed by a treat-and-extend protocol. Patients who remained quiescent 12 weeks from their prior treatment entered a treatment pause and were switched to *pro re nata* (PRN) treatment (based on vision, clinical exam, and/or imaging studies). Proteomic analysis was performed on aqueous fluid to identify proteins that correlate with patients’ response to treatment.

**Results:**

At the end of 1 year, 38 of 122 eyes (31%) entered a treatment pause (≥30 weeks). Conversely, 21 of 122 eyes (17%) failed extension and required monthly treatment at the end of year 1. Proteomic analysis of aqueous fluid identified proteins that correlated with patients’ response to treatment, including proteins previously implicated in AMD pathogenesis. Interestingly, apolipoprotein-B100 (ApoB100), a principal component of drusen implicated in the progression of nonneovascular AMD, was increased in treated patients who required less frequent injections. ApoB100 expression was higher in AMD eyes compared with controls but was lower in eyes that develop choroidal neovascularization (CNV), consistent with a protective role. Accordingly, mice overexpressing ApoB100 were partially protected from laser-induced CNV.

**Funding:**

This work was supported by the National Eye Institute, National Institutes of Health grants R01EY029750, R01EY025705, and R01 EY27961; the Research to Prevent Blindness, Inc.; the Alcon Research Institute; and Johns Hopkins University through the Robert Bond Welch and Branna and Irving Sisenwein professorships in ophthalmology.

**Conclusion:**

Aqueous biomarkers could help identify patients with nvAMD who may not require or benefit from long-term treatment with anti-VEGF therapy.

## Introduction

Neovascular (nv) age-related macular degeneration (AMD) is the leading cause of severe vision loss in elderly Americans (1). If left untreated, patients with nvAMD suffer dramatic and irreversible vision loss from edema, bleeding and scarring caused by growth of abnormal leaky blood vessels (i.e., choroidal neovascularization or CNV). The recent introduction of therapies targeting vascular endothelial growth factor (VEGF), has had a remarkable impact on patients with nvAMD (2). VEGF is a potent endothelial mitogen and permeability factor that is regulated by the transcription factor hypoxia-inducible factor 1 (HIF-1) (3–5). Several multi-centered, randomized, controlled clinical trials have demonstrated that a minority of patients with nvAMD treated with anti-VEGF therapy lose further vision, with up to half experiencing a clinically significant (i.e., 3 line or more on the Early Treatment Diabetic Retinopathy Study [ETDRS] chart) improvement in vision (6). However, anti-VEGF therapies are provided indefinitely, raising concerns about the substantial economic and social burden of frequent clinic visits for elderly patients who often require assistance for transportation and mobility (7). While this has prompted the development of longer-acting anti-VEGF therapies, which require less frequent injections (8), there are also concerns about ocular risks and theoretical concerns about the safety associated with frequent, indefinite intraocular injections with anti-VEGF therapies (9, 10). Given rising health care costs, an aging population, and the anticipated increase in the number of patients with nvAMD worldwide, the sustainability of indefinite intraocular injections with anti-VEGF therapy is a reasonable concern. This concern has prompted some clinicians to seek to reduce the number of treatments with currently available therapies (and, in turn, injection/treatment-related adverse events) and the number of visits. This may be one explanation for why the success of anti-VEGF therapies, when translated to real-world clinical practice, has been less impressive than that observed in clinical trials (11). Investigators are therefore exploring alternative approaches that refine initial treatment protocols without sacrificing the visual acuity benefits observed with monthly or bimonthly treatment.

One approach many clinicians have adapted to reduce the treatment burden is to monitor patients with nvAMD using a fixed interval, but to only treat patients as needed or *pro re nata* (PRN). This approach has the potential to reduce the total annual number of injections but would not impact either the frequency of patient visits or the number of imaging studies performed. This reactive approach presumes to capture most relapses promptly while minimizing the number of treatments. In the CATT study, PRN treatment with ranibizumab was demonstrated to be noninferior to monthly treatment but reduced the number of injections by more than a third (from 24 to 15) by the end of year 2; similar results were observed with PRN treatment with bevacizumab (12). These results were corroborated in the HARBOR study (13). However, with a fixed treatment interval, the number of patient visits was the same in patients treated monthly and those treated PRN.

An alternative approach to optimize the efficacy of a drug for each patient while minimizing the number of injections is to treat-and-extend (TAE), a strategy in which the response of an individual patient to treatment is used to determine whether the interval between treatments can be extended for that particular patient. This approach provides mandatory dosing but on a personalized schedule. TAE is a proactive approach that assumes that patients manifest a regular pattern of disease activity (i.e., a patient’s response to their previous injection can predict their response to a subsequent injection). The TREND study demonstrated that the TAE approach was non-inferior to monthly dosing with ranibizumab and resulted in a decreased mean number of injections by 22% (11.1 vs. 8.7) and visits by 21% (11.2 vs. 8.9) in the first year compared with monthly dosing (14). Similar results were observed in the CANTREAT study, which also demonstrated non-inferiority for TAE with ranibizumab compared with monthly treatment and reduced the number of injections by 25% (from 23.5 to 17.6) compared with monthly treatment over 2 years (15). Both studies also demonstrated that up to a quarter of patients fail attempts to extend the interval between treatments, requiring monthly injections. While half of patients in the TREND study required treatment every 4 to 8 weeks despite using the TAE approach, approximately 20% of patients with nvAMD could be extended to 12 weeks or more between injections, at which point they received a maintenance treatment. TAE therefore can reduce annually both the total number of visits as well as the total number of injections. However, TAE may still result in overtreating patients during the extension phase and may be treating patients unnecessarily during the maintenance phase.

Here we use a hybrid of the PRN and TAE approaches, which we describe as treat-and-extend-pause/monitor (TEP/M; Supplemental Figure 1), to assess whether we can safely and effectively wean patients with nvAMD off anti-VEGF therapy. We then categorized patients based on the interval between treatments required to adequately manage their disease by the end of 1 year and performed proteomic analyses of aqueous fluid (obtained at the time of treatment initiation) comparing patients who require monthly treatment with those who required less frequent treatment. Using this unbiased approach, we set out to identify proteins that predict or directly contribute to the response of patients with nvAMD to treatment with anti-VEGF therapy.

## Results

### Ascertainment of study eyes of primary cohort.

A review of charts of patients from the clinic of a single vitreoretinal surgeon at a satellite office of a tertiary care center from 2013 to 2020 identified 207 eyes from insured patients with a diagnosis of nvAMD (diagnosed by clinical exam and SD-OCT and confirmed by fluorescein angiography) who underwent intravitreal injections with anti-VEGF therapy (Table 1 and Supplemental Figure 2; supplemental material available online with this article; https://doi.org/10.1172/JCI144469DS1). Inclusion and exclusion criteria are described in Methods. Patients included in the study agreed to participate in the TEP/M approach and were followed after initiating treatment without interruption for 1 year (102 eyes; 87 patients) and at 2 years (65 eyes; Table 1 and Supplemental Figure 2).

### Outcome measurements at 12 and 24 months during steady-state phase of TEP/M protocol.

Following the TEP/M protocol, the mean change in central subfield thickness (CST) on SD-OCT was –79.3 ± 9.6 and the mean change in vision was 2 ± 2 letters at the end of year 1 (Table 2). The percentage of patients with a mean improvement or decline in vision of 5 letters or greater was 46% and 19%, respectively. The mean interval between treatments (using a maximal interval of 6 months for patients who were weaned from treatment) was 11.3 ± 0.7 weeks by the end of year 1. Accordingly, the mean number of treatments received was 7.8 ± 0.2. Compared with traditional monthly treatment, the number of treatments using the TEP/M protocol was reduced by 40% (from 13.0 to 7.8) at 12 months.

There were 65 eyes that were followed under the TEP/M protocol for at least 2 years without deviations from protocol, as described for year 1. The mean interval between treatments using a maximal interval of 6 months for patients who were weaned from treatment, was 14.6 ± 1.1 weeks after 24 months (Table 3). In turn, the mean number of treatments received by the end of year 2 was 12.2 ± 1.1. Compared with traditional bimonthly treatment (following 3 initial monthly treatments), the number of treatments using the TEP/M protocol was reduced by 13% (from 14.0 to 12.2) at 24 months (Table 3). Compared with traditional monthly treatment, the number of treatments using the TEP/M protocol was reduced by 51% (from 25.0 to 12.2) at 24 months.

### Patients successfully weaned from anti-VEGF therapy using TEP/M approach.

Using the TEP/M protocol, we were able to successfully wean off treatment (i.e., patients not requiring treatment on 3 consecutive scheduled visits, and for at least 30 weeks from their last injection) 31% (32/102) of patients within the first year (Supplemental Table 1 and Table 4). By the end of year 2, 38% (25/65) of patients were weaned from treatment. Of the 22 patients successfully weaned off treatment who were followed for a minimum of 2 years, 73% (16/22) remained off treatment at the end of year 2 and 87% (13/15) of patients weaned off treatment by the end of year 2 remained off treatment at the end of year 3 (Supplemental Table 2). Of the 102 eyes followed in this group, 43% (44/102) were successfully paused (30 weeks or greater) during the course of their follow up (Supplemental Table 3; median length of follow up 28.5 months, range 12 to 72 months).

### Patients requiring maintenance therapy versus patients weaned from anti-VEGF therapy.

A comparison of eyes that required maintenance treatment every 8 to 12 weeks (*n =* 35 eyes) with those who were successfully weaned off treatment by year 1 (*n =* 32 eyes) demonstrated that the mean number of treatments received was higher in the maintenance group compared with the weaned group (7.8 ± 0.3 vs. 6.0 ± 0.3; *P <* 0.0001; Supplemental Table 4), as expected. The mean change in CST was similar for these 2 groups (–80.6 ± 18.6 vs. –70.4 ± 13.3; *P =* 0.861). However, the percentage of these patients with a 5-or-more-letter improvement was lower in the maintenance group compared with the weaned group (34.3% vs. 53.1%; *P =* 0.007; Supplemental Table 4). The percentage of these patients with a 5-or-more-letter decrease in vision was similar (17.1% vs. 18.8%; *P =* 0.713). When we specifically examined the subset of 8 eyes that were weaned from treatment but subsequently had recurrent disease activity, we observed a mean vision loss of 6 ± 2 letters at the time of their recurrence (Supplemental Table 5). Following reinitiation of treatment with anti-VEGF therapy, these patients recovered 3 ± 2 letters after a single retreatment. This suggested that weaned patients who experience a recurrence of CNV remain sensitive to anti-VEGF therapy and recover vision upon resuming treatment (Supplemental Table 5).

Overall, adverse outcomes (endophthalmitis, vitreous hemorrhage, retinal tear, retinal detachment, RPE tear, subretinal hemorrhage) were uncommon. There was no increase in adverse outcomes in patients with nvAMD receiving anti-VEGF therapy every 4 to 6 weeks compared with those receiving treatment every 8 to 12 weeks at 24 or 36 months (Supplemental Table 6). There was no increase in adverse outcomes in patients successfully weaned off treatment compared with patients requiring maintenance treatment (every 4 to 6 weeks or every 8 to 12 weeks; Supplemental Table 6).

### Confirmatory cohort of patients with nvAMD treated with TEP/M.

Collectively, these results suggest that the TEP/M approach can safely and effectively wean up to 30% of patients with nvAMD off anti-VEGF therapy in 12 months. To determine if the results observed in this retrospective analysis of patients with nvAMD can be extrapolated to other patient populations, we examined a second, independent cohort of patients treated using the same TEP/M protocol. To this end, we reviewed charts of a separate cohort of patients from a tertiary hospital-based clinic between 2013 to 2020 and identified 32 eyes from insured patients with a diagnosis of nvAMD (using same criteria as primary cohort by clinical exam and SD-OCT and confirmed by fluorescein angiography) who underwent intravitreal injections with anti-VEGF therapy (Supplemental Figure 3 and Table 5). Inclusion/exclusion criteria were identical to that used for the primary cohort. Patients included in this second independent cohort also agreed to participate in the TEP/M approach and were followed after initiating treatment without interruption for at least 1 year. In this second, independent cohort, 30% (6/32) of eyes were successfully weaned from anti-VEGF therapy (i.e., patients not requiring treatment on 3 consecutive scheduled visits, and for at least 30 weeks from their last injection) by the end of year 1 (Table 5), similar to what was observed in the initial cohort of patients.

### Response of fluid on OCT to anti-VEGF therapy using TEP/M in weanable versus nonweanable patients.

**Figure 1:**
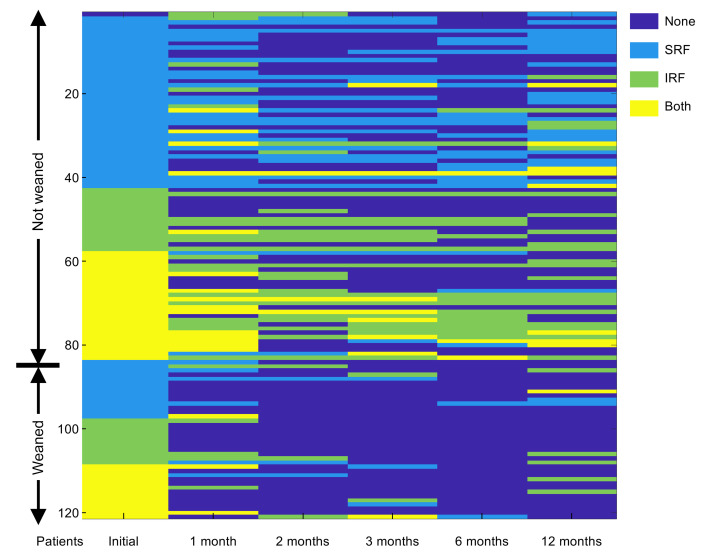
Heatmap comparing fluid over time for eyes of patients who required sustained anti-VEGF treatment versus those who were successfully weaned from anti-VEGF therapy by 12 months. OCT images were obtained from all 102 eligible patients who underwent the TEP/M approach for at least 12 months. Presence of fluid on OCT was graded independently by 2 investigators for the presence of no fluid, subretinal fluid (SRF), intraretinal fluid (IRF), or both at time points 0, 1, 2, 3, 6, and 12 months after initiation of protocol. Fluid status overtime for each individual patient is graphically represented with dark blue denoting no fluid; light blue, SRF; light green, IRF; and yellow, both. Patients were grouped into 2 categories: those not weaned (requiring sustained treatment every 4–12 weeks) and those weaned off treatment. Within each group, patients were sorted by severity of fluid (none < SRF < IRF < both).

We next set out to examine whether we could distinguish between patients who required frequent or maintenance therapy from those who were ultimately weaned from treatment. To this end, SD-OCT images from patients in both the initial and the confirmatory TEP/M cohorts were graded prior to initiation of treatment, at the time of diagnosis (presentation), and at 1, 2, 3, 6, and 12 months after initiating treatment for the presence of fluid. Each SD-OCT was independently classified as having no fluid (none), subretinal fluid (SRF), intraretinal fluid (IRF), or SRF and IRF (both) by 2 independent masked graders; disagreements were reconciled by a third grader. Patients were grouped based on frequency of treatment at 12 months to compare those who were able to successfully be weaned from treatment with those who were unable to be weaned from treatment (Figure 1). Interestingly, while the distribution of fluid was similar in both groups at presentation, 63% (24/38) of weanable patients had complete resolution of fluid after their first treatment compared with only 30% (25/84) of nonweanable patients (Table 6). The percentage of nonweanable patients without fluid peaked after 2 treatments at under 50%. Conversely, 84% (32/38) of weanable patients had no fluid at month 2 and 95% (36/38) had no fluid by month 6 (Table 6).

### Aqueous VEGF levels fail to predict response of nvAMD to anti-VEGF therapy.

**Figure 2:**
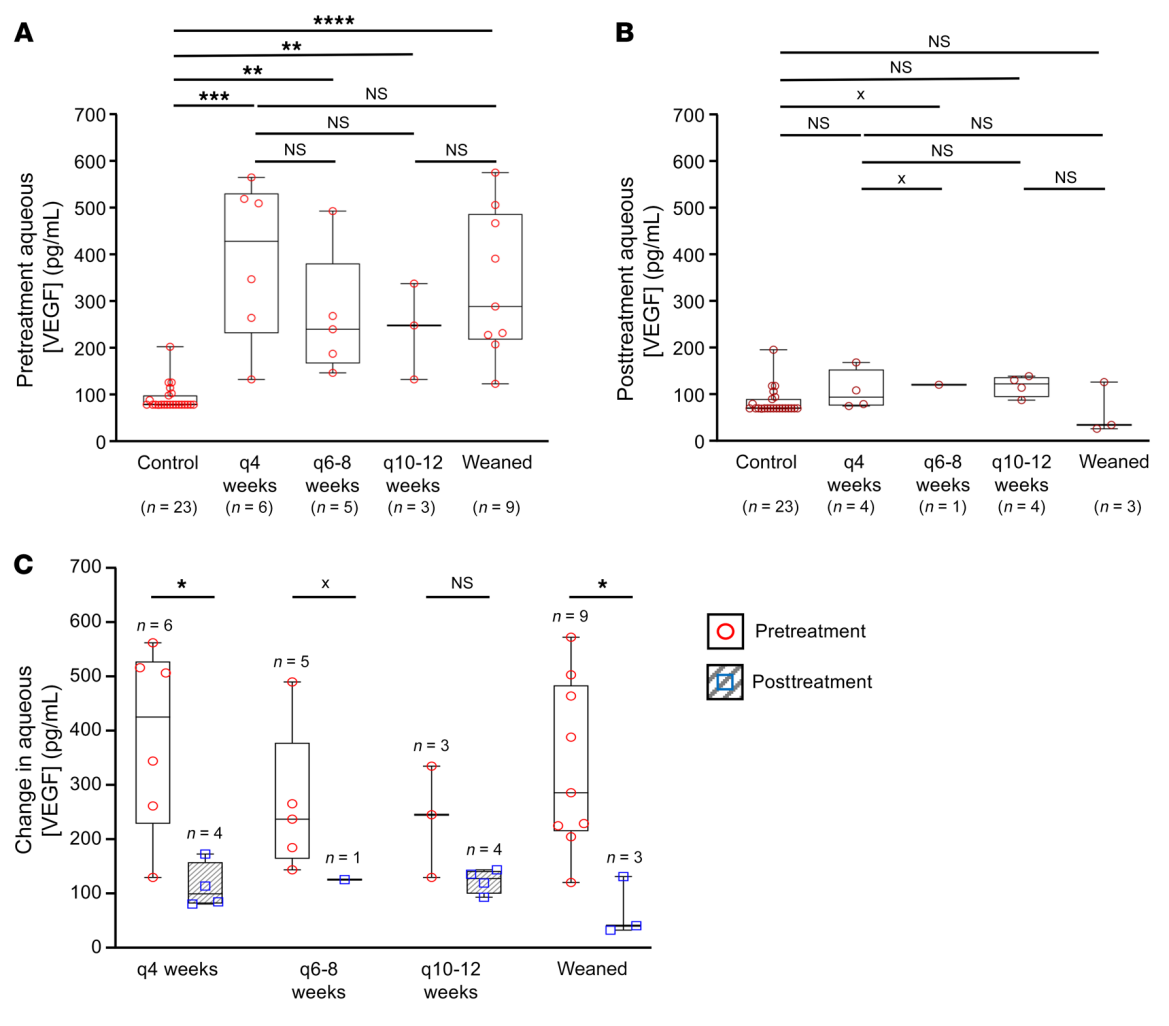
Aqueous levels of VEGF in TEP/M patients. (**A**) Pretreatment aqueous VEGF levels (prior to first injection) for patients with increasing interval between treatments at 12 months (from subset of TEP/M patients). (**B**) Posttreatment aqueous VEGF levels (having received their first injection) for patients with increasing interval between treatments at 12 months (from subset of TEP/M patients). (**C**) Comparison of pretreatment and posttreatment aqueous VEGF levels for patients with increasing interval between treatments at 12 months (from subset of TEP/M patients). Statistical analysis was performed using Wilcoxon rank sum test. *P < 0.05; **P < 0.01; ***P < 0.001; ****P < 0.0001. An x indicates that statistical analyses could not be performed due to insufficient samples.

These results suggest that the early response to treatment by anti-VEGF therapy may predict whether patients could ultimately be weaned from treatment. In this regard, emerging therapies under development are being designed to reduce VEGF levels more effectively (16, 17). We therefore sought to determine whether aqueous VEGF levels predict the response of patients with nvAMD to treatment with current anti-VEGF therapies. A subset of patients included in our study consented to provide aqueous samples on presentation, prior to initiation of treatment with anti-VEGF therapy (Supplemental Table 7). The concentration of VEGF in these aqueous samples was measured by ELISA in patients who required treatment intervals either monthly (every 4 weeks), every 6 to 8 weeks, or every 10 to 12 weeks, or in patients who were weaned from treatment at the end of year 1. Compared with control patients, we observed a marked increase in the pretreatment aqueous VEGF levels in all 4 groups of patients with nvAMD (Figure 2A). However, we did not observe a difference in VEGF levels among these 4 groups (Figure 2A). Since we observed a significant difference in intraretinal and subretinal fluid on SD-OCT in weanable compared with nonweanable patients as early as 1 month after their first treatment, we next examined whether the VEGF levels were different in patients after initiating treatment with anti-VEGF therapy. The posttreatment aqueous levels of VEGF within their first 3 months of initiating anti-VEGF therapy was similar to control levels and did not differ among the 4 subgroups of patients with nvAMD (Figure 2B). Similarly, the decline in aqueous VEGF levels following initiation with anti-VEGF therapy also did not correlate with the response to treatment with anti-VEGF therapy (Figure 2C). Collectively, these results suggest that aqueous VEGF levels do not predict the response of patients with nvAMD to treatment with anti-VEGF therapy.

### Proteomic analysis to identify aqueous-associated proteins from patients with nvAMD.

**Figure 3:**
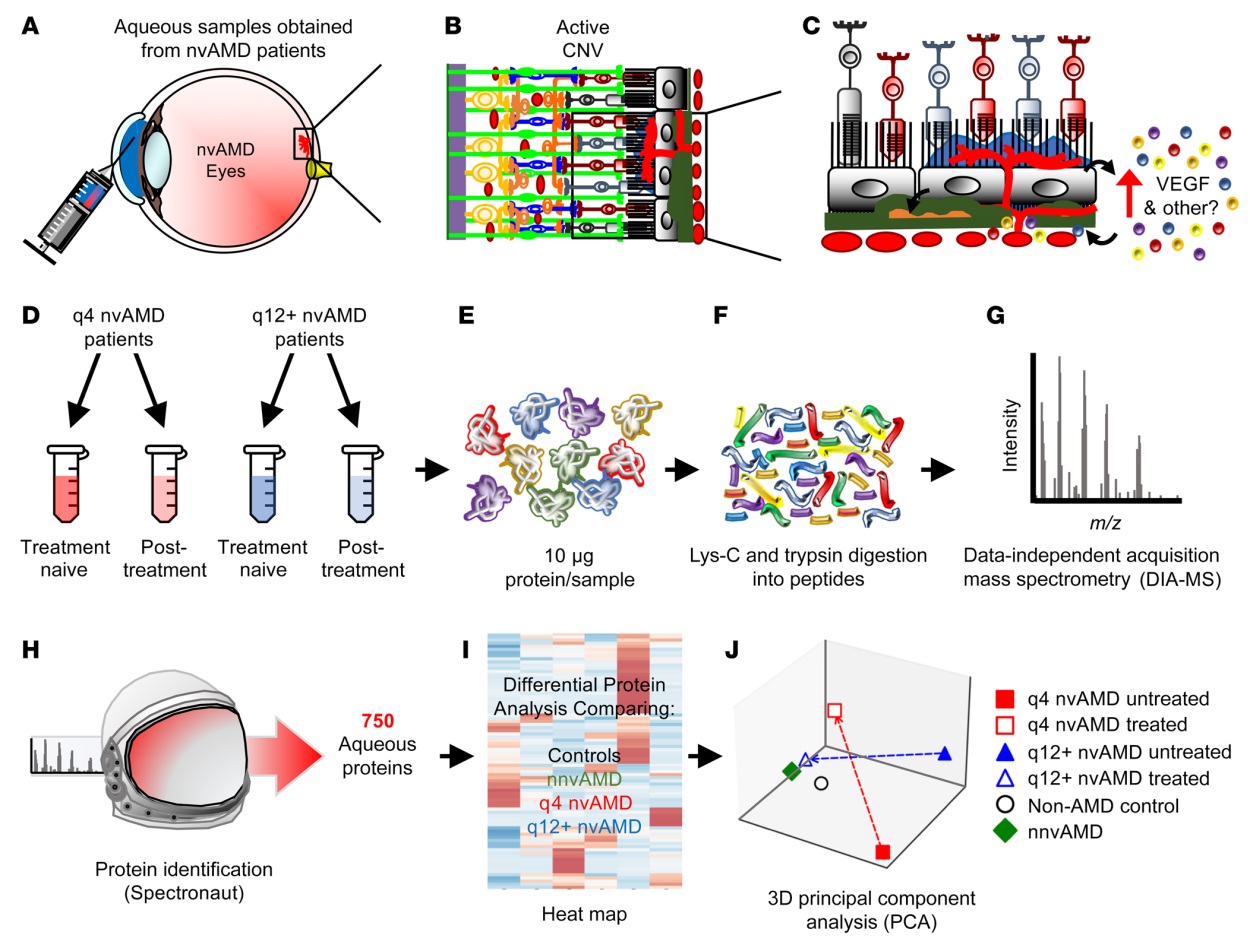
Overview of patient sample collection and proteomics screen in untreated (anti-VEGF naive) and treated (those who have received anti-VEGF therapy) patients with wet AMD. (**A**) Aqueous samples were collected from clinic patients with nvAMD via anterior chamber paracentesis. (**B** and **C**) Illustration of disrupted outer blood-retinal barrier in nvAMD (**B**) and release of VEGF and other angiogenic mediators that promote choroidal neovascularization (**C**). (**D**) Patient samples were separated into those that required monthly (q4) treatment versus those who were able to be extended to 12 or more weeks (q12+) and by anti-VEGF treatment status (treatment naive and after treatment). (**E**) Pooled patient aqueous samples containing 10 μg of protein were prepared. (**F**) Samples were digested by proteolytic enzymes. (**G**) Mass spectrometry was performed to analyze each sample. (**H**) Unique proteins were identified using the Spectronaut Proteomics System. (**I**) Differential protein analysis was performed comparing q4 and q12+ nvAMD patient samples with non-AMD controls and patients with (dry) nnvAMD. (**J**) To evaluate the molecular effects of anti-VEGF treatment in patients, we utilized principal component analysis (PCA). The samples used in the analysis include q4 and q12+ patients and their responses to anti-VEGF treatment, as well as non-AMD controls and patients with nnvAMD. The first 3 components were selected for the 3D PCA.

We next sought whether other aqueous-associated protein(s) could be identified to help predict or contribute to the response to treatment with anti-VEGF therapy (Figure 3, A–C). To this end, we took an unbiased approach and examined aqueous fluid from TEP/M patients using proteomics (Figure 3, D–G). Due to the limited volume of aqueous fluid available from TEP/M patients, and to reduce the influence of outliers, we performed proteomic analyses on pooled aqueous samples from untreated patients with nvAMD who required monthly treatment (q4 untx group = nonweanable; *n =* 3) with those patients with nvAMD who required treatment every 12 weeks or could be weaned from treatment (q12+ untx group = weanable; *n =* 7) at the end of year 1.

The OCT analysis of fluid in weanable versus nonweanable TEP/M patients demonstrated no difference in the presence or absence of fluid prior to the initiation of treatment, but a measurable difference was detectable as early as 1 month after a single treatment with anti-VEGF therapy. This suggested that the difference in behavior between these 2 groups can be detected early after treatment initiation. We therefore also performed a proteomic analysis from pooled samples from treated patients (following their mandatory monthly first, second, or third treatment) who required monthly treatment (q4 tx group; *n =* 6) compared with those who required treatment every 12 weeks or could be weaned from treatment (q12+ tx group; *n =* 5) at the end of year 1.

We detected 750 proteins in the aqueous fluid from the 4 pooled samples (Figure 3H). A heat map was generated comparing proteins expressed in these 4 groups, as well as in control patients and patients with dry (nnv)AMD (Figure 3I and Supplemental Figure 4A). Principal component analysis demonstrated a closer relationship between protein levels in controls and patients with nnvAMD compared with the 2 untreated groups of patients with nvAMD (Figure 3J). Following treatment, the q12+ tx group was more similar to control and nnvAMD groups than was the q4 tx group (Figure 3J, Supplemental Figure 4, B–D, and Supplemental Table 8).

### Protein families increased or decreased in aqueous fluid from weanable versus nonweanable patients with nvAMD.

**Figure 4:**
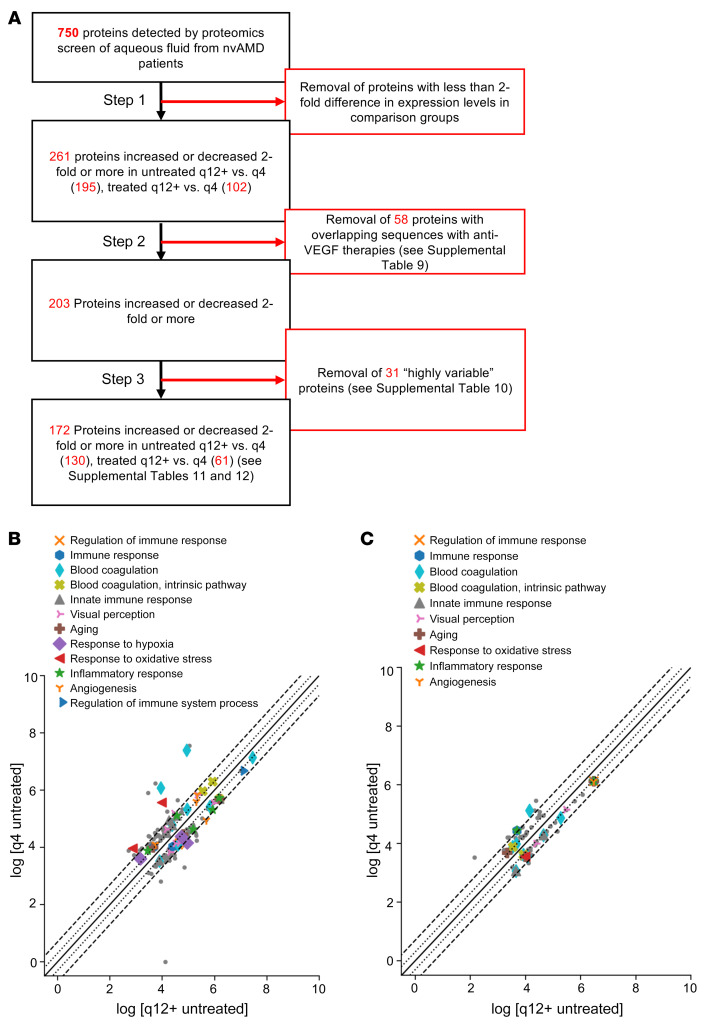
Stepwise identification and isolation of key proteins driving phenotype of patients with nvAMD in untreated and treated q12+ versus q4 groups. (**A**) Flow diagram describing the process of removing proteins with similar expression levels, those with sequences that overlapped with anti-VEGF therapies, and those that were highly variable between q4 and q12+ groups to identify proteins of interest. (**B**) Scatter plot of identified aqueous proteins with 2-fold changes between q4 untreated patients and q12+ untreated patients. (**C**) Scatter plot of identified aqueous proteins with 2-fold changes between q4 treated patients and q12+ treated patients. In **B** and **C**, colored markers represent enriched biological process from the proteomics analysis; gray dots are the proteins with no enriched biological processes.

To determine which among the 750 aqueous proteins could be used as aqueous-associated proteins that may predict the response of patients with nvAMD to anti-VEGF therapy, we compared the expression levels of these proteins in the 2 pairs of patients. Comparison of these pairs identified 261 proteins that were increased or decreased 2-fold or more in either untx or q12+ tx groups compared with the untx or q4 tx groups (Figure 4A, Step 1, and Supplemental Figure 5). Fifty-eight proteins (Supplemental Table 9) had sequences that overlapped with the sequence of currently available anti-VEGF therapies (i.e., aflibercept, bevacizumab, or ranibizumab). This left 203 proteins that were increased or decreased 2-fold or more in the q12+ compared with the q4 groups (Figure 4A, Step 2).

To exclude proteins that were increased or decreased due to chance (i.e., due to the variable expression levels of the protein in the aqueous of patients with nvAMD, regardless of their response to anti-VEGF therapy), we performed a proteomics analysis on a separate cohort of untreated patients with nvAMD that were arbitrarily divided into 2 groups (nvAMD1, *n =* 9; nvAMD2, *n =* 9). When we compared the proteins between these 2 groups, we observed 31 proteins (Supplemental Table 10 and Supplemental Figure 6C) that were increased or decreased 2-fold or greater in nvAMD1 compared with nvAMD2 and that were also identified in the comparison between q4 and q12+ groups. These 31 proteins were therefore designated highly variable proteins and removed from the q4 versus q12+ analyses, leaving 172 proteins that were increased or decreased 2-fold or greater in the comparison between q4 versus q12+ aqueous samples (Step 3 in Figure 4A). Gene ontology analyses of q4 versus q12+ untx (Figure 4B) and tx (Figure 4C) demonstrated that proteins that were increased or decreased 2-fold or more (Supplemental Tables 11 and 12) fell into several categories, including aging, angiogenesis, blood coagulation, immune response, and response to wound healing, hypoxia, and oxidative stress, all previously implicated in the pathogenesis of nvAMD.

### Identification of proteins that may contribute to the response of nvAMD eyes to treatment with anti-VEGF therapy.

To further narrow down the proteins identified in the proteomics screen to those proteins that may serve as biomarkers or contribute to the development or progression of nvAMD, we next examined which proteins were differentially expressed in untreated patients with nvAMD (*n =* 18) compared with control patients (*n =* 24). We observed 263 proteins that were increased or decreased 2-fold or greater in untreated patients with nvAMD compared with control patients (Supplemental Figure 6A). To examine whether these proteins were differentially expressed in nvAMD eyes compared with control eyes (rather than variably expressed in control eyes), we arbitrarily divided a group of control patients into 2 groups (control 1, *n =* 12; control 2, *n =* 12) and compared the expression of aqueous proteins using proteomics. Using this approach, we noted that almost half (over 47%) of proteins detected in the arbitrarily divided control groups were increased or decreased 2-fold or greater between these 2 groups (Supplemental Figure 6B). This was significantly greater than the diversity of protein expression observed in patients with nvAMD (in which 11% of proteins were increased or decreased 2-fold or greater between the 2 groups; Supplemental Figure 6C). This suggested that proteomic analyses of aqueous fluid comparing patients with nvAMD — and, by extension, any other disease group — with a control group can be influenced by the significant variability of protein levels among controls and may therefore not be helpful in identifying proteins specifically increased in patients with nvAMD.

**Figure 5:**
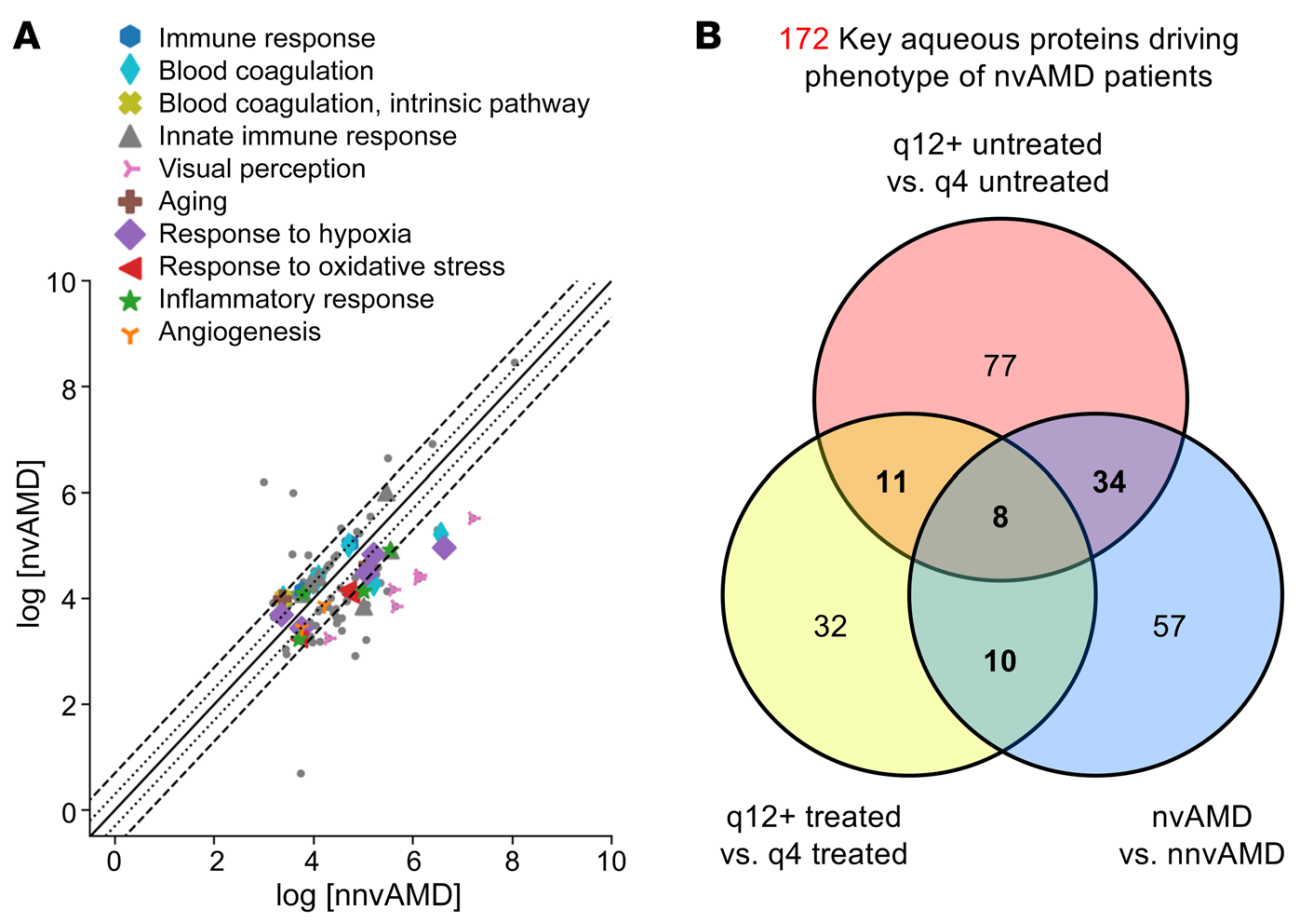
Comparison of expression of key proteins in nvAMD versus nnvAMD and identification of overlapping proteins. (**A**) Scatter plot of identified aqueous proteins with 2-fold changes between patients with (dry) nnvAMD and those with (wet) nvAMD. Colored markers represent enriched biological process from the proteomics analysis; gray dots are the proteins with no enriched biological processes. (**B**) Venn diagram describing overlapping proteins identified in the comparisons between q4 versus q12 untreated patients, q4 versus q12 treated patients, and patients with nnvAMD versus those with nvAMD (see Supplemental Table 12).

We instead compared the expression of proteins in patients with nvAMD with patients with nnvAMD (*n =* 18) and identified 109 proteins that were differentially expressed (by at least 2-fold) in nnvAMD versus nvAMD and may therefore contribute to the progression of dry to wet AMD (Supplemental Figure 6D). Gene ontology analyses revealed the same categories identified in the comparison between q4 and q12+ groups (Figure 5A). Indeed, among the 109 proteins identified in the nvAMD versus nnvAMD comparison, 42 overlapped with proteins identified in the q12+ untx group compared with the q4 untx group, 18 overlapped with proteins identified in the q12+ tx group compared with the q4 tx group, and 8 proteins were identified in all 3 comparisons (Figure 5B and Supplemental Tables 13 and 14).

### Complement proteins differentially expressed in aqueous fluid from weanable versus non-weanable patients with nvAMD.

**Figure 6:**
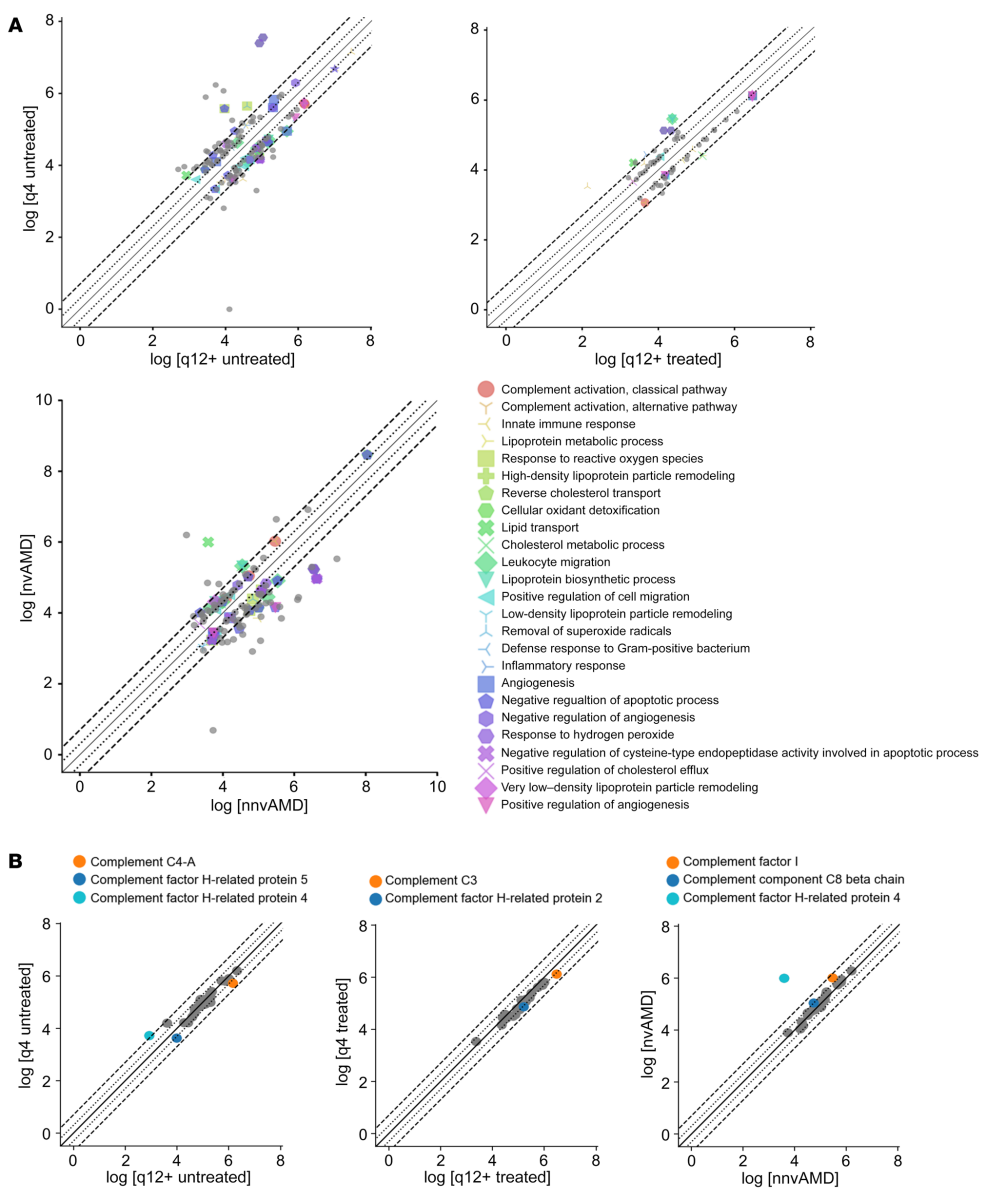
Comparison of expression of key immunomodulatory proteins and complement proteins. (**A**) Comparison of the expression of aqueous proteins identified in the proteomics analyses between q4 versus q12 untreated patients, q4 versus q12 treated patients, and patients with nnvAMD versus those with nvAMD, highlighting immunomodulatory proteins. Proteins increased or decreased less than 2-fold were excluded from this analysis. (**B**) Comparison of the expression of aqueous proteins identified in the proteomics analyses between q4 versus q12 untreated patients, q4 versus q12 treated patients, and patients with nnvAMD versus those with nvAMD,s highlighting complement proteins.

Recent data strongly implicate immune dysregulation in the development of AMD (18). Genome-wide association studies have identified several variants of the innate immune system complement genes that influence the risk of developing AMD (19). Among the proteins that were increased or decreased 2-fold or more in q4 versus q12+ groups were several immunomodulatory proteins (Figure 6A). This included 6 complement-related proteins: 4 that were increased or decreased 2-fold or greater in the q12+ untx group compared with the q4 untx group and 2 that were increased or decreased 2-fold or greater in the q12+ tx group compared with the q4 tx group (Supplemental Table 15). One of these complement-related proteins was identified as highly variable (Supplemental Table 10), leaving 5 complement-related proteins: C3, complement factor 4-A (C4-A), complement Factor H-related (CFHR) protein 2, CFHR 4, and CFHR 5 (Figure 6B and Supplemental Table 15). In the comparison of nnvAMD versus nvAMD, we identified 3 additional complement-related proteins that were increased or decreased 2-fold or greater: complement factor 1, complement component C8 beta chain, and CFHR 4.

### Proteins that respond differently in aqueous fluid from weanable versus nonweanable patients with nvAMD.

**Figure 7:**
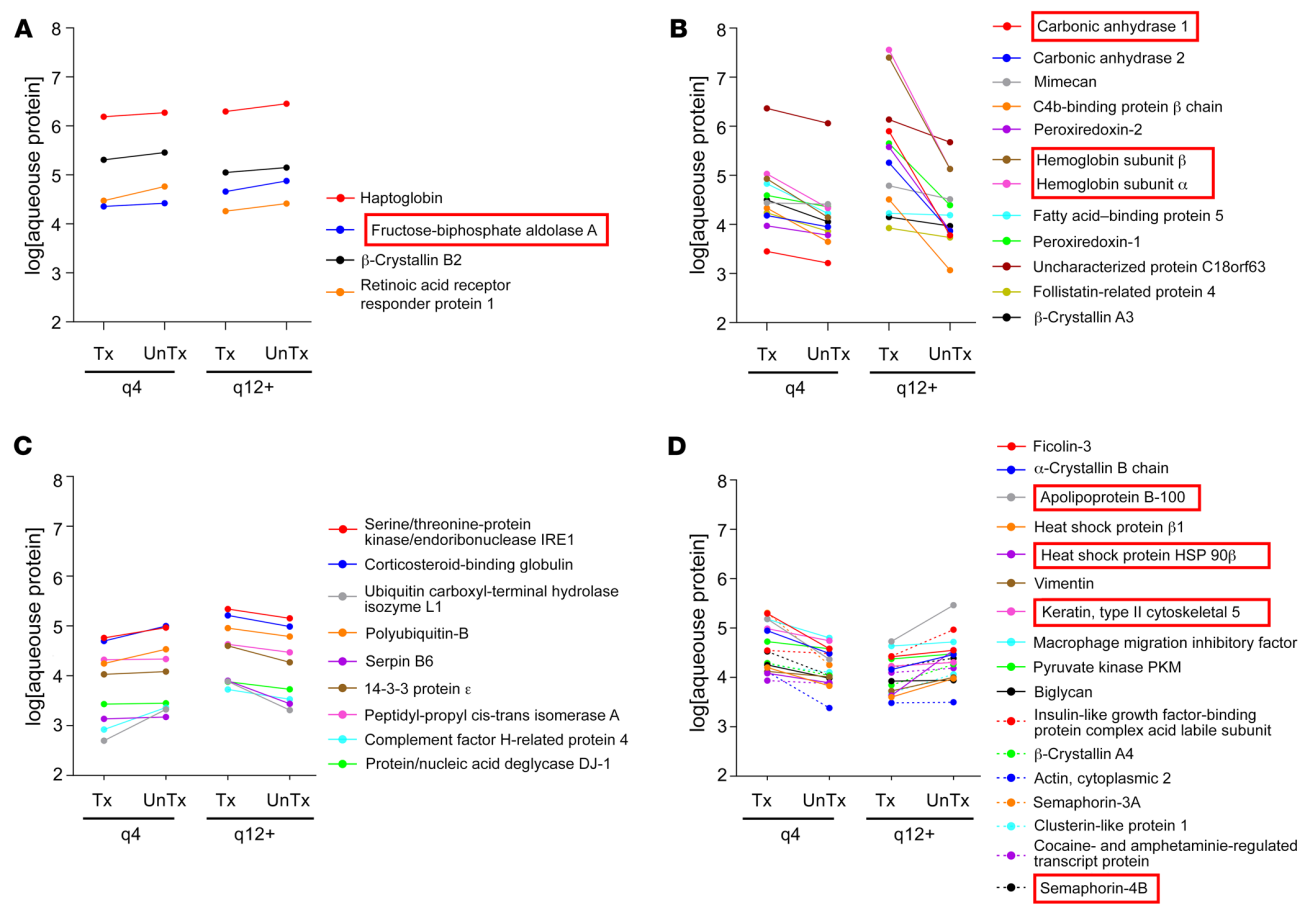
Key proteins identified by proteomics analyses and their response to anti-VEGF treatment in q4 versus q12 patients. (**A**) Proteins that increased following treatment with anti-VEGF therapy in both q4 and q12+ patients with nvAMD. (**B**) Proteins that decreased following treatment with anti-VEGF therapy in both q4 and q12+ patients with nvAMD. (**C**) Proteins that increased following treatment with anti-VEGF therapy in q4 patients with nvAMD but decreased following treatment in the q12+ patients with nvAMD. (**D**) Proteins that decreased following treatment with anti-VEGF therapy in q4 patients with nvAMD but increased following treatment in the q12+ patients with nvAMD. Red box highlights proteins present in all 3 comparisons (q4 vs. q12+ untreated; q4 vs. q12+ treated, and nvAMD vs. nnvAMD; see Figure 5B).

As stated above, the OCT analysis of fluid in weanable versus nonweanable TEP/M patients demonstrated no difference in the presence or absence of fluid prior to the initiation of treatment, but a measurable difference was detectable as early as 1 month after a single treatment with anti-VEGF therapy. We therefore set out to identify proteins that respond differently in q4 versus q12+ patients following initiation with treatment of anti-VEGF therapy. To this end, we compared the expression of proteins identified in the proteomic analyses in the q4 untx group with the q4 tx group and the q12+ untx group with the q12+ tx group (Figure 7, A–D). Interestingly, while 16 of the 42 proteins identified in all 3 proteomics screens (q4 vs. q12+ untreated; q4 vs. q12+ treated; and nvAMD vs. nnvAMD) behaved similarly in both the q4 and q12+ groups (Figure 7, A and B), 9 proteins increased in the q4 group but decreased in the q12+ group in response to treatment (Figure 7C) while 17 proteins decreased in the q4 group but increased in the q12+ group in response to treatment (Figure 7D), suggesting that they may directly contribute to the different response of q4 versus q12+ patients with nvAMD to anti-VEGF therapy. Of these 26 proteins, 4 were among the 8 proteins differentially expressed (2-fold or more) in all 3 comparisons (q4 vs. q12+ untreated; q4 vs. q12+ treated; and nvAMD vs. nnvAMD; see Figure 5B).

### Apolipoprotein B-100 plays a protective role in the development of CNV.

**Figure 8:**
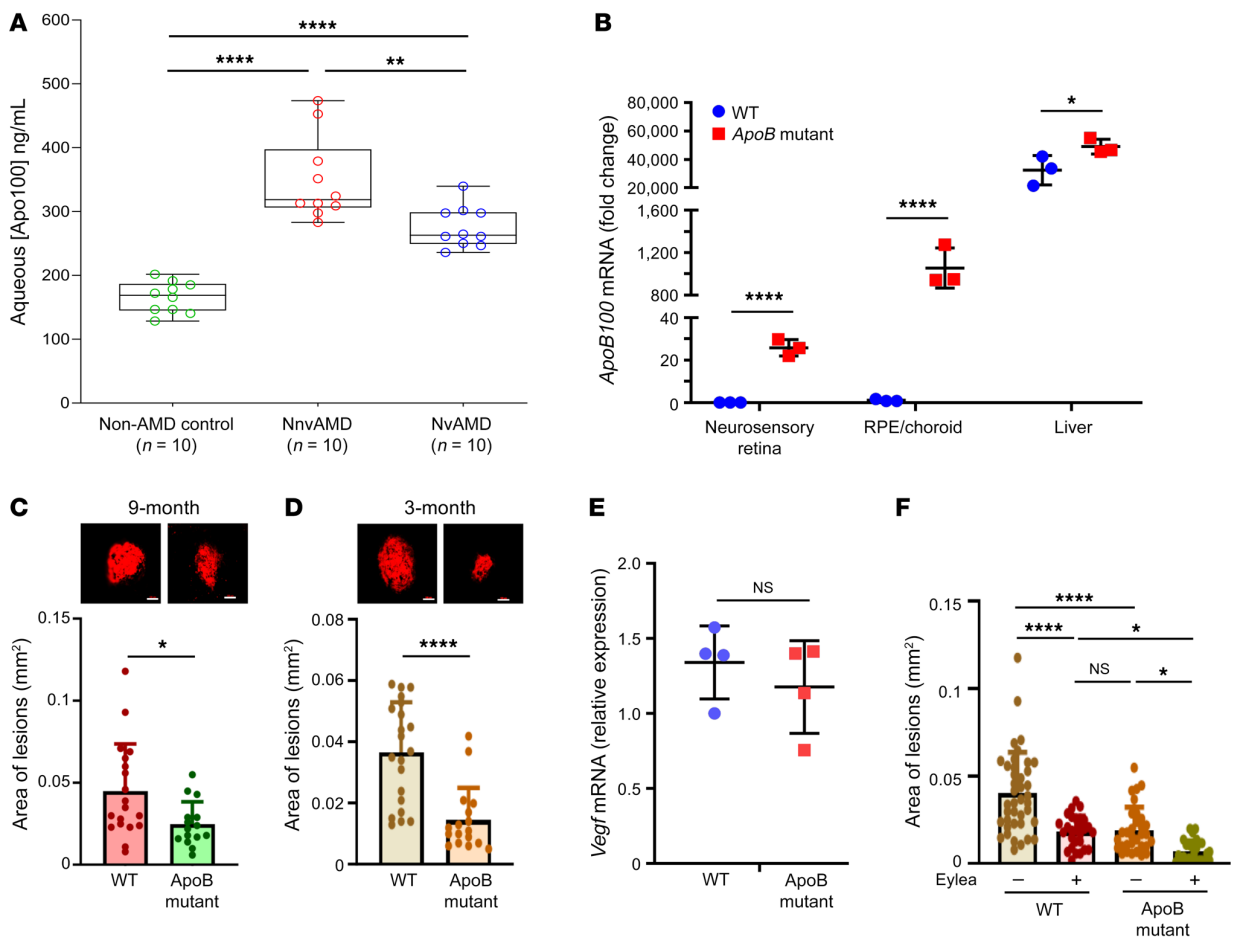
Apolipoprotein B-100 plays a protective role in the development of CNV. (**A**) ApoB100 protein level in aqueous of non-AMD control, nnvAMD, and nvAMD. Compared with that of non-AMD control, ApoB100 was significantly higher in the aqueous of patients with nnvAMD and nvAMD. Moreover, ApoB100 levels in the aqueous of patients with nnvAMD was dramatically higher than that in patients with nvAMD. (**B**) *ApoB100* mRNA level in neurosensory retinal, RPE/choroid, and liver of both WT and *ApoB* mutant mice. *ApoB*100 levels in all 3 tissues of *ApoB* mutant mice are significantly higher than those from WT mice. (**C **and** D**) Laser CNV lesion size in 9-month-old (**C**) and 3-month-old (**D**) WT and ApoB100 mutant mice 7 days after treatment with laser. Scale bars: 100 μm. Individual dots represent CNV spots from choroids of 4 mice in each case. A reduction in choroidal neovascularization was observed in ApoB100 transgenic mice compared with WT mice. Results were plotted as mean ± SD. Unpaired Student’s *t* test was used to compare the 2 groups. *P < 0.05. (**E**) *Vegf* mRNA level in RPE/choroid from WT and *ApoB* mutant mice. Results were plotted as mean ± SD. *P* values were generated by 2-tailed Student’s *t* test. (**F**) Laser CNV comparison between WT and *ApoB* mutant mice 7 days following treatment with laser. A subset of mice were treated with 200 ng aflibercept on day 3 following laser treatment. *n =* 4 to 8 animals for each condition. Results were plotted as mean ± SD. One-way ANOVA with Tukey’s multiple comparison test was used to compare the different treatments with each other. ****P < 0.0001; ***P < 0.001; **P < 0.01; *P < 0.05; and NS, *P* > 0.05.

Among the 4 proteins that responded differently in the q4 compared with q12+ groups following treatment, and that were present in all 3 comparisons (q4 vs. q12+ untreated; q4 vs. q12+ treated, and nvAMD vs. nnvAMD) was apolipoprotein-B100 (ApoB100). ApoB100 expression decreased in the q4 group but increased in the q12+ group following treatment (Figure 7D). ApoB100 has been reported to be secreted by the retinal pigment epithelium (20–24), and accumulates within Bruch’s membrane as an early component of drusen (25–29). It is speculated that oxidized ApoB100 may contribute to the development of (dry) nnvAMD and the progression to (wet) nvAMD (30). However, our results demonstrate a paradoxical increase in ApoB100 in the eyes of patients who respond better to anti-VEGF therapy. To corroborate the results from our proteomics studies, we measured by ELISA the aqueous ApoB100 levels in an unrelated cohort of patients with nnvAMD, untreated nvAMD, and non-AMD control patients (Supplemental Table 16). We observed an increase in aqueous ApoB100 levels in patients with early and intermediate nnvAMD compared with control patients (Figure 8A), consistent with a role for ApoB100 in the development of dry AMD. The aqueous ApoB100 levels in patients with nvAMD was similarly increased compared with controls. However, aqueous ApoB100 levels were significantly higher in patients with early and intermediate nnvAMD compared with patients with nvAMD (Figure 8A), suggesting that ApoB100 levels decrease with progression from dry to wet AMD.

Based on the observation that aqueous ApoB100 levels were increased in treated patients with nvAMD who required infrequent treatment compared with those who required monthly treatment, as well as the reduced aqueous ApoB100 levels in patients with nvAMD compared with those with early and intermediate nnvAMD, we hypothesized that expression of ApoB100 may play a protective role in nvAMD. To interrogate this hypothesis, we took advantage of a homozygous mutant mouse line in which a CTA to TTA mutation was introduced to sequences corresponding to the ApoB48 editing codon (codon 2179) in exon 26, resulting in a marked reduction of ApoB48 expression (31). Expression of ApoB in these mice therefore is shifted from 50% ApoB100 and 50% ApoB48 to over 90% ApoB100. qPCR analysis demonstrated a marked increase in the mRNA expression of the *ApoB100* isoform in the RPE/choroid from ApoB mutant mice compared with WT mice (Figure 8B). Aged (9-month-old) ApoB mutant animals were subjected to laser treatment and the size of CNV lesions was measured 7 days later. We observed smaller CNV lesions in the *ApoB* mutant mice compared with WT mice (Figure 8C), consistent with a protective role for ApoB100 in nvAMD.

Although their development is normal, *ApoB* mutant mice have lower HDL cholesterol levels than WT mice. Over time this, in turn, leads to lipoprotein accumulation in Bruch’s membrane (20). To examine whether the effects on CNV lesion size in *ApoB* mutant mice was due to expression of ApoB100 in the RPE/choroid rather than to lipid deposition and alterations of Bruch’s membrane, we examined the development of CNV lesions in younger animals and observed a similar reduction in CNV lesion size in 3-month-old *ApoB* mutant mice compared with control WT mice (Figure 8D).

We next examined whether increased expression of ApoB100 influenced VEGF expression or the response of CNV lesions to anti-VEGF therapy. To this end, we first examined the expression of *Vegf* mRNA by qPCR in the RPE/choroid from *ApoB* mutant mice compared with WT mice. We observed similar levels of *Vegf* mRNA in both *ApoB* mutant and WT mice (Figure 8E). We next treated *ApoB* mutant mice with a single intraocular injection with aflibercept (200 ng) at day 3 following laser treatment. Examination of animals at day 7 demonstrated further reduction in the size of the CNV lesion in *ApoB* mutant mice, similar to WT control mice (Figure 8F). CNV lesions were smaller in *ApoB* mutant mice treated with aflibercept compared with WT mice treated with aflibercept (Figure 8F). Collectively, these data suggest that ApoB100, an early and key component of drusen, could play an unexpected protective role in the development of CNV in patients with nvAMD, and that this role may be independent of *Vegf* mRNA expression and additive to anti-VEGF therapy.

## Discussion

It is anticipated that the number of people who have AMD will approach 300 million by 2040 (32). Close to 14 million people in the United States have AMD (33), a number that increases by almost 300,000 every year (34). Despite affecting only 10% of patients with AMD, CNV is the cause of severe vision loss in 90% of patients with AMD (35), and remains the leading cause of blindness among the elderly in the United States (36). The introduction of anti-VEGF therapies has had a remarkable impact on patients with nvAMD who previously suffered major, irreversible vision loss from edema, bleeding, or scarring caused by CNV (2). Although calculating the economic impact of preventing vision loss — or improving vision — in treated patients is challenging, there is little doubt that anti-VEGF therapy has improved the quality of life of millions of patients worldwide. Current efforts are now focused on efforts to refine treatment protocols to reduce the number of injections (and treatment-related adverse events) without sacrificing the visual acuity benefits observed with monthly or bimonthly treatment.

Prior clinical trials have demonstrated that the TAE approach was not inferior to monthly treatment with anti-VEGF therapy, but reduced the number of treatments by 25% in 2 years (15). Several subsequent studies have shown similar success using TAE, with the benefit of reducing patient visits and injection burden, while preserving visual outcomes (14, 37–40). It has also been reported that switching from PRN to TAE results in an improvement in both CST and visual acuity (41, 42). However, the requirement for maintenance therapy in patients with nvAMD remains under debate.

Here, we use a hybrid of the TAE and PRN approaches, TEP/M, to optimize the efficacy of anti-VEGF therapy for each specific patient, while minimizing the number of treatments needed. Compared with traditional bimonthly treatment (following 3 initial monthly treatments), TEP/M resulted in a 27% decrease in the mean number of treatments after 2 years (10.2 ± 0.9 vs. 14). Compared with traditional monthly treatment, the number of treatments using the TEP/M protocol was reduced by 44% (25 vs. 14.0 ± 0.8) at 2 years. These results demonstrate that TEP/M effectively reduces the number of treatments and extends the interval between visits for anti-VEGF therapy. Using the TEP/M protocol, we further observed that 31% of patients with nvAMD (32/102) could be weaned off treatment within 1 year. Retreatment of patients was required in only 27% of patients (6/22) in the second year following initiation of the treatment pause. By the end of year 2, 38% of patients with nvAMD (25/65) had been weaned off treatment, with only 13% of patients (2/15) requiring retreatment in their third year. This is a dramatic departure from current treatment strategies which require indefinite treatment for nvAMD. We corroborated these findings in a second independent cohort of patients and observed similar results.

Two prior reports have previously evaluated a hybrid TAE/PRN approach to wean patients with nvAMD off treatment (43, 44). In the first study, patients receiving anti-VEGF therapy underwent a TAE protocol once the macula was determined to be dry on SD-OCT (43), similar to our study. Although this prior report did not utilize a strict protocol for treatment extension, it was reported that the interval between visits was extended by 1 or 2 weeks if there was no fluid on SD-OCT. Once patients were extended to 12 weeks, they received treatment, followed by 2 additional maintenance treatments 12 weeks apart (for almost 9 months) before they were taken off treatment (after a minimum of 8 injections over approximately 16 months). Using this approach, 143 of 385 eyes (37.3%) were successfully weaned off treatment. This is similar to what we observed in the present study after 1 year. However, in the prior study, the time interval required to successfully wean these patients off treatment was not reported. Additionally, the authors included in the “successfully weaned off treatment” group any patient who was taken off treatment at any time during the study, over the entire follow-up period, and no criteria were set to exclude patients who were nonadherent with their regimen or follow-up visits.

A more recent study also examined the potential of weaning patients with nvAMD off treatment with anti-VEGF therapy using a TAE protocol (44). While the investigators began extending the interval between visits after the second treatment, the interval plateaued at 16 rather than 12 weeks, and was followed by 3 additional maintenance treatments 16 weeks apart (i.e., for almost 1 year) before holding treatment and entering a monitoring phase during which patients were examined every 3 to 4 months. The exit criteria were reached by 17% (100/598 eyes) of eyes. These patients required a minimum of 10 injections over 2 years with an average treatment duration of 4.5 years (range of 2–7 years).

These studies included broad inclusion criteria for patient selection, with unclear adjustments for prior treatment, comorbid ischemic retinopathies, or other underlying disease. While both studies utilized SD-OCT at all initial and follow-up visits, fluorescein angiogram (FA) was not required for confirmation of diagnosis in one study (44). Importantly, patients who did not adhere to regular treatment intervals were still included in their analyses (43, 44).

Limitations of the clinical portion of our study include that it is a retrospective study with a limited number of patients, the follow-up is 1 year (with a subset of patients extended out for 2 or 3 years), and best corrected visual acuity (BCVA) was not obtained at each visit. Nonetheless, in our study, diagnosis of nvAMD by clinical exam and SD-OCT was confirmed by FA. And patients with a second diagnosis that could influence their response to treatment were excluded from the study. Moreover, our results were corroborated in a second independent cohort of patients. We also required strict adherence to the TAE protocol; significant (defined) deviations from the protocol led to exclusion of the patient from our analysis. We did not include any maintenance therapy period, allowing us to rapidly wean patients off treatment within 8 months of treatment initiation and with as few as 6 injections compared with 8 to 10 injections over 16 to 24 months (43, 44). Despite this rapid weaning approach with our TEP/M protocol, 31% of all patients were successfully weaned off treatment by 1 year. This rapid approach is supported by our OCT analysis which demonstrated a clear distinction in the response to treatment between nonweanable and weanable patients as early as 1 month after treatment. Interestingly, the CST was similar in patients weaned from treatment compared with patients who required maintenance doses (every 8 to 12 weeks). Moreover, more patients weaned from treatment experienced a 5-or-more-letter improvement in vision compared with patients requiring maintenance therapy. There was no difference in adverse outcomes in patients weaned off treatment compared with patients receiving treatment every 4 to 6 weeks or every 8 to 12 weeks. And patients who were weaned from treatment and subsequently had a recurrence of CNV remained sensitive to resuming treatment with anti-VEGF therapy. Collectively, these results demonstrate the safety and efficacy of the TEP/M approach.

These studies have important implications for the management of patients with nvAMD. The potential long-term cost savings for patients with nvAMD successfully weaned off treatment will have an important impact on cost-effective analyses of different anti-VEGF therapies. Moreover, our results suggest that up to one-third of patients with nvAMD may not require or benefit from the anticipated introduction of second-generation, longer-acting anti-VEGF therapies (Supplemental Figure 7 and ref. 45). This is particularly important given emerging concerns of the untoward consequences of long-term suppression of VEGF in the eyes of patients with AMD (10).

Our results further suggest that patients with nvAMD are not a homogeneous population, but instead are comprised of 3 distinct subgroups (Supplemental Figure 7): patients who respond inadequately despite monthly anti-VEGF therapy (q4 patients; approximately 20%); patients who respond to anti-VEGF therapy, but require indefinite treatment (approximately 50%); and patients who can be weaned off treatment (q12+ patients; approximately 30%). These subgroups can be distinguished by their early response to treatment, as reflected by their SD-OCT images. Moreover, we demonstrate that pre- and posttreatment aqueous VEGF levels could not predict which patients will require monthly treatment compared with those patients who could be extended to 6 to 12 weeks or weaned from treatment. These results suggest that additional factors may influence how patients with nvAMD respond to anti-VEGF therapy.

These findings also have important clinical implications for patients with nvAMD. They suggest that there are definable subgroups of patients with nvAMD who respond differently to anti-VEGF therapy. It is also not known whether specific anti-VEGF therapies may be more effective than others at weaning patients off therapy. The ability to wean patients off therapy may have a significant impact on the long-term cost-effectiveness of anti-VEGF therapies. These unanswered questions are under current investigation. This is particularly relevant given emerging ethical issues about the distribution of cost-effective versus most effective anti-VEGF therapies among patients with ocular neovascular disease (46).

The ability to determine how patients will respond to anti-VEGF therapy will also be essential for deciding which patients will benefit from the next generation of longer-acting anti-VEGF therapies versus those who may be successfully (and transiently) treated with currently available therapies. In the second half of this study, we therefore took an unbiased approach to identify aqueous biomarkers that may help distinguish between (or contribute to) these subgroups of patients with nvAMD. Using proteomics, we identified 172 proteins that were differentially expressed in the aqueous of untreated or treated q12+ compared with q4 patients. Gene ontology analyses demonstrated that these proteins fell into several families, including aging, angiogenesis, blood coagulation, immune response, and response to wound healing, hypoxia, and oxidative stress, all previously connected with AMD pathogenesis. These results suggest that aqueous levels of a subgroup of specific proteins could serve as biomarkers, which may help predict which patient would benefit from new longer-acting therapies or therapies targeting additional vasoactive mediators. Future studies will be needed to identify which of these proteins that could comprise an array of biomarkers would help predict how patients with nvAMD will respond to current and future therapies. Of note, our proteomic analysis of non-AMD control patients demonstrated remarkable variability within this group. This suggests that caution should be taken when using aqueous fluid from control patients in proteomic analyses.

Many of the proteins identified in the proteomic analyses have previously been implicated in the pathogenesis of AMD, suggesting that they may further play a functional role in determining which patients respond adequately to anti-VEGF therapy. In this regard, among the proteins we identified that were differentially expressed in our proteomic analyses were 8 complement-related proteins. The complement system plays an important role in the pathogenesis of AMD (19). Drusen, present in all patients with AMD, are comprised of proteins involved in inflammation and include components of the complement system (30). There is strong genetic evidence for an association of variants in the genes of the complement system, including C3 and CFH and the development of advanced AMD (19). While patients with AMD exhibit elevated serum levels of activation products of complement proteins, the role of these complement proteins in the development of AMD remains unclear. One of the 8 complement-related proteins identified in our proteomics studies was C3. C3 was 2-fold higher in patients requiring monthly treatment with anti-VEGF therapy compared with those requiring less frequent treatment. This observation is consistent with prior studies suggesting that C3 activation may contribute to AMD pathogenesis.

Of the remaining 7 complement-related proteins identified in our proteomics studies, 3 were members of the CFHR family of proteins. Variants of the complement inhibitor CFH have also been reported to increase the risk for developing AMD (47). CFHR proteins are evolutionarily and structurally related to CFH and have previously been implicated in other diseases involving complement dysregulation (48). However, the CFHR proteins do not appear to share CFH’s role as a negative complement regulator. Rather, recent evidence suggests that CFHR proteins may instead enhance complement activation. CFHR4 was more than 6-fold lower in patients requiring monthly treatment with anti-VEGF therapy compared with those requiring less frequent treatment. Conversely, CFHR2 and CFHR5 were 2-fold higher in patients requiring monthly treatment with anti-VEGF therapy compared with those requiring less frequent treatment. Whether CFHR proteins contribute to the development of nvAMD, or the response of patients with nvAMD to anti-VEGF therapy, requires further investigation.

We further identify 8 proteins differentially expressed (2-fold or more) in all 3 comparisons (q4 vs. q12+ untreated; q4 vs. q12+ treated; and nvAMD vs. nnvAMD). These proteins included hemoglobin subunits α and β, carbonic anhydrase 1, fructose-bisphosphate aldolase A, keratin, type II cytoskeletal 5, ApoB100, HSP 90ß, and semaphorin-4B. Among these 8 proteins, the latter 4 proteins responded differently in the q4 compared with q12+ groups following treatment with anti-VEGF therapy, suggesting that they may influence the response of patients with nvAMD to treatment. Among these proteins, ApoB100 is of particular interest in AMD pathogenesis. ApoB100 has been reported to be secreted by the retinal pigment epithelium (20–24), and accumulates within Bruch’s membrane as an early component of drusen (25–29). It is speculated that oxidized ApoB100 may contribute to the development of (dry) nnvAMD and the progression to (wet) nvAMD (30). However, our results suggest ApoB100 may also play a novel and paradoxical protective role in (wet) nvAMD. We observed that aqueous levels of ApoB100 were higher in patients with early and intermediate nnvAMD compared with control patients, but lower in patients with nvAMD compared with patients with nnvAMD. These observations suggest that increased ApoB100 expression correlates with the development of nnvAMD, but lower ApoB100 levels correlate with nvAMD. Indeed, we observed that mutant mice overexpressing ApoB100 in the RPE/choroid developed smaller CNV lesions in the laser-induced CNV model compared with WT controls. Moreover, the influence of ApoB100 on CNV lesions was independent of *Vegf* mRNA expression and additive to anti-VEGF therapy.

Collectively, these observations implicate ApoB100, a key component of drusen, in protection against the development of nvAMD. In light of these unexpected findings, it may be prudent to revisit the role of drusen, or components of drusen, in the pathogenesis of AMD. In this regard, we also observed differential expression of apolipoprotein A and apolipoprotein D in treated patients with nvAMD who required monthly treatment with anti-VEGF therapy compared with those requiring less frequent treatment. Future studies may help determine whether (and how) these lipoproteins, as well as the other proteins identified in these proteomics studies, contribute to the development of nnvAMD vs. nvAMD, or to the response of patients with nvAMD to anti-VEGF therapy.

## Methods

### Clinical studies.

Please see the Supplemental Material for details of clinical studies.

### Aqueous samples.

Institutional Review Board approval from the Johns Hopkins University School of Medicine (Baltimore, MD) was obtained for all patient samples used in this HIPAA-compliant study. Aqueous samples (0.1–0.2 mL) were collected via limbal paracentesis using a 30-gauge needle attached to a tuberculin syringe from consenting patients at the Wilmer Eye Institute immediately after performing intravitreal injection for active CNV. Consent was written and voluntary without stipend. Aqueous samples were immediately processed and stored at –80°C prior to analysis.

### ELISA.

Human VEGF (Duoset) and ApoB100 (Quantikine) ELISA kits were purchased from R&D Systems. Aqueous samples were analyzed for VEGF and ApoB100 (10 μL of aqueous diluted 1:10) using ELISAs, and performed according to the manufacturer’s protocols. All ELISAs were performed in duplicate (VEGF) or triplicate (ApoB100) and quantitation was performed using the standard curve constructed with the standards included in the kit.

### Proteomics.

Please see the Supplemental Material for details of proteomics analyses.

### Animal studies.

Please see the Supplemental Material for details of animal studies.

### Statistics.

Categorical variables were presented as percentages and compared using the 2-sided χ^2^ test with significance set at *P* less than 0.05. Patients’ BCVAs were converted from Snellen Visual Acuity into logarithm of the minimum angle of resolution (logMAR) score for statistical analysis. LogMAR was converted into approximate ETDRS scores to determine mean gains or losses of letters at 12 and 24 months compared with their initial vision prior to treatment initiation. Data for continuous variables were recorded as mean ± SEM. Assuming nonparametric data, an unpaired, 2-tailed, Mann-Whitney test analysis with significance set at *P* less than 0.05 was used to compare mean data points in this study. Scatterplots for VEGF and ApoB100 were generated using MATLAB. For the animal studies, statistical differences between 2 heterogenous groups were determined by unpaired Student’s *t* test and for more than 2 groups were determined by 1-way ANOVA with Turkey’s multiple comparison test. Statistical analyses were performed using Microsoft Excel and GraphPad Prism 8.0 software (GraphPad). One-way ANOVA was used to perform statistical testing for age difference of patients in the proteomics analysis, VEGF ELISA, and ApoB100 ELISA assays. The Wilcoxon rank sum test was used for statistical analysis between patient groups in pre- and posttreatment VEGF ELISAs. Adjusted *P* values were calculated using the FDR method to account for multiple comparisons. Fisher’s exact test was used to perform statistical testing for sex and pseudophakic difference. Both 1-way ANOVA and Fisher’s exact test were carried out in R environment (4.0.5). Statistical significance was defined as *P* less than 0.05. *P < 0.05; **P < 0.01; ***P < 0.001; ****P < 0.0001.

### Study approval.

Institutional review board approval from the Johns Hopkins University School of Medicine was obtained for all patient information, including OCT images and aqueous samples, used in this study. All experiments involving animals were performed in accordance with the Association for Research in Vision and Ophthalmology Statement for the Use of Animals in Ophthalmic and Vision Research and formally reviewed and approved by the Johns Hopkins University IACUC on Animal Research Reporting.

## Author contributions

AS was the primary contributor to research design. XC, JCS, AD, CG, TPP, ZY, LC, YW, DM, KJ, YJD, and AS were responsible for research execution and data acquisition. XC, JCS, AD, CG, TPP, MWH, JQ, HZ, SM, and AS were the primary contributors to data analysis and interpretation. AS prepared the manuscript and revisions were provided by XC, JCS, MWH, JTH, JQ, HZ, and SM. AS had full access to all data in the study and takes responsibility for the integrity of the data and the accuracy of the data analysis.

## Supplementary Material

Supplemental data

ICMJE disclosure forms

## Figures and Tables

**Table 6:**
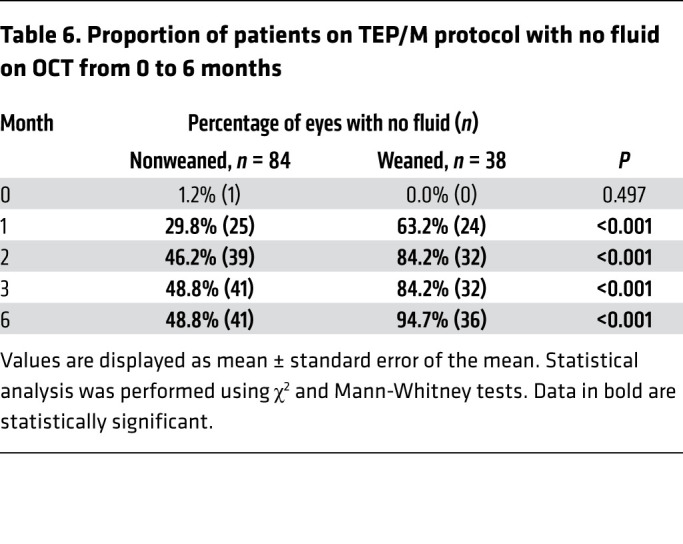
Proportion of patients on TEP/M protocol with no fluid on OCT from 0 to 6 months

**Table 5:**
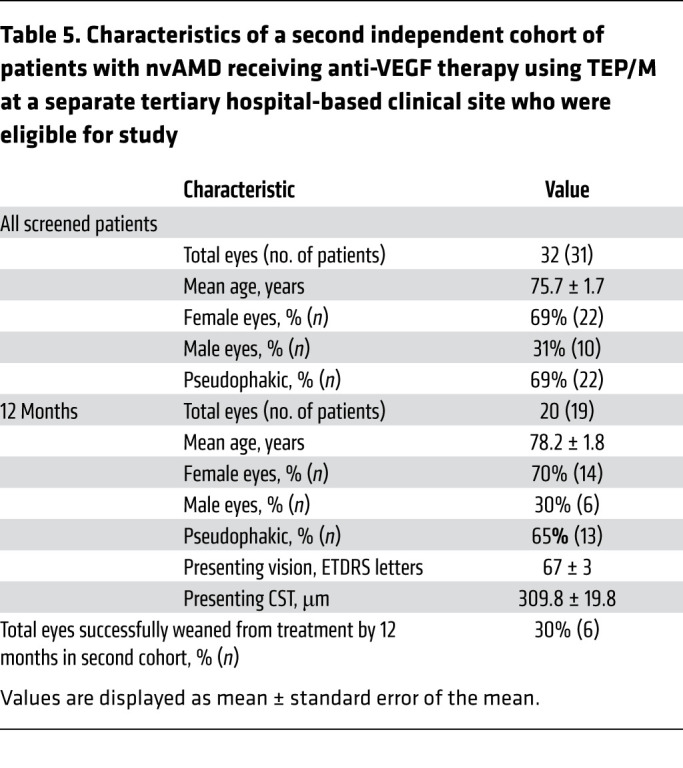
Characteristics of a second independent cohort of patients with nvAMD receiving anti-VEGF therapy using TEP/M at a separate tertiary hospital-based clinical site who were eligible for study

**Table 4:**
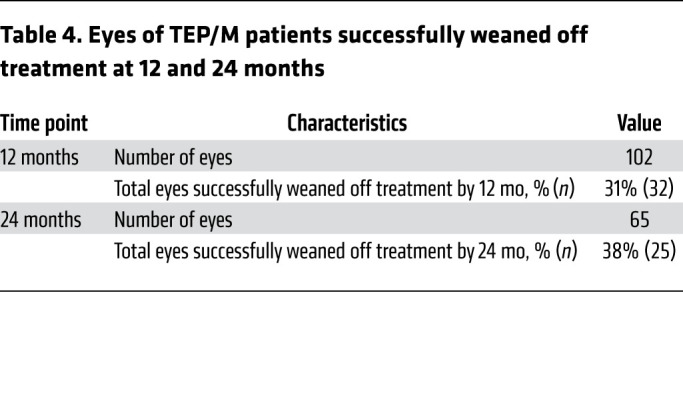
Eyes of TEP/M patients successfully weaned off treatment at 12 and 24 months

**Table 3:**
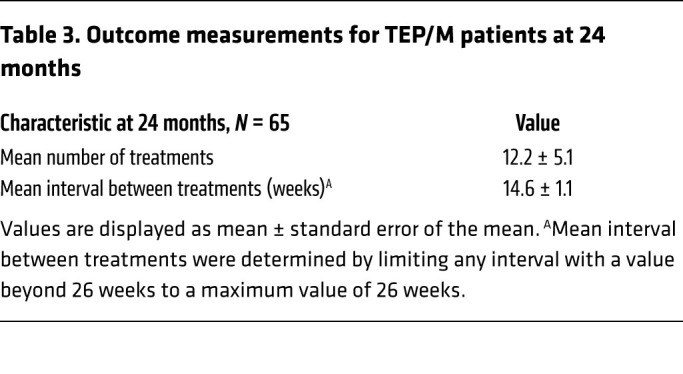
Outcome measurements for TEP/M patients at 24 months

**Table 2:**
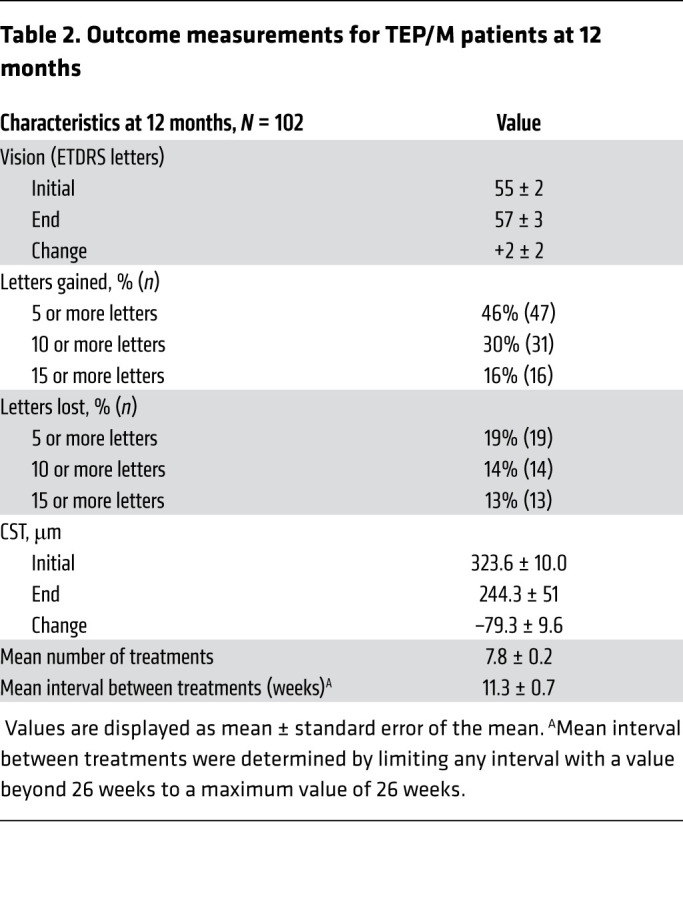
Outcome measurements for TEP/M patients at 12 months

**Table 1:**
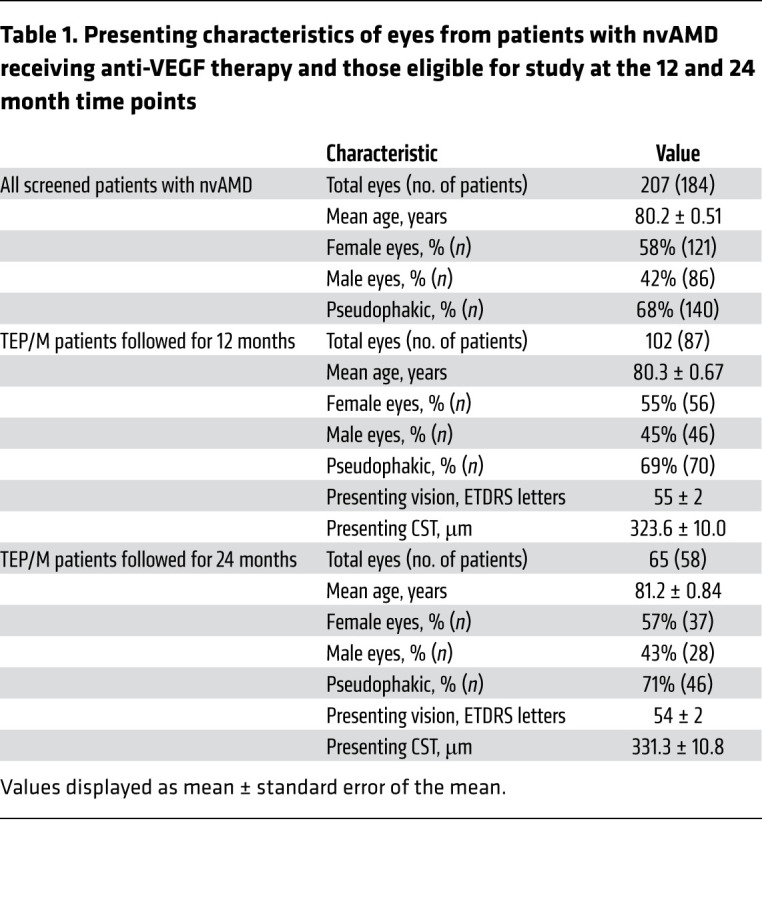
Presenting characteristics of eyes from patients with nvAMD receiving anti-VEGF therapy and those eligible for study at the 12 and 24 month time points

## Version 1 12/07/2021

In-Press Preview

## Version 2 01/18/2022

Electronic publication
