# Supplementary material for: Aqueous proteins help predict the response of patients with neovascular age-related macular degeneration to anti-VEGF therapy
Source: J Clin Invest. 2022 Jan 18;132(2):e144469. doi: 10.1172/JCI144469 (PMC8759792; doi:10.1172/JCI144469)

Clinical Studies

Patients

Insured patients at a satellite office (primary cohort) or a hospital-based clinic (secondary cohort) of a tertiary care center who were diagnosed with nvAMD were treated with bevacizumab, aflibercept, or Lucentis (hereafter, "anti-VEGF therapy"). Inclusion criteria included a new diagnosis of nvAMD [diagnosed on clinical exam and Spectral Domain Optical Coherence Tomography (SD-OCT), and confirmed by fluorescein angiography] or newly active nvAMD (with an absence of activity or treatment for at least 1 year and a presenting vision of 20/400 or better) who were followed after initiating treatment without interruption for at least 1 year. All patients included in this study agreed to participate in the "treat-and-extend-pause/monitor" (TEP/M) approach (see below). Exclusion criteria included an underlying ischemic retinal disease at the time of diagnosis (e.g., diabetic retinopathy, retinal vein occlusion, sickle cell retinopathy), other forms of choroidal neovascularization (e.g., presumed ocular histoplasmosis, polypoidal choroidopathy, etc.), post-surgical cystoid macular edema, active ocular inflammation, or a recent (within 1 year) history of intraocular steroid treatment. Patients in whom treatment was withheld after being determined to be "futile" (i.e., the presence of atrophy or a disciform scar involving the foveal center at presentation which prevented an improvement in vision despite an adequate response to treatment of fluid) were excluded from the study. Patients who had a delayed return for a scheduled treatment by more than 2 weeks once in the first 3 months or twice in the first year were also excluded from the study.

Treat-and-Extend-Pause/Monitor (TEP/M) Protocol

All patients included in this study underwent a TEP/M approach in which patients receiving anti-VEGF therapy underwent 3 consecutive monthly injections followed by a TAE approach in which the interval between treatments was extended by 2 weeks at each visit in the absence (or near absence) of fluid, hemorrhage, or a decline vision (Supplemental Figure 1). Vision, clinical exam, and SD-OCT were obtained at all scheduled clinic visits. Return of fluid or a decline in vision resulted in a reduction of the interval between visits by 2-4 weeks; new hemorrhage would result in a decrease in the interval between visits back to 1 month. Stable patients extended to 12 weeks had their treatment held (paused), at which point the patients entered the "PRN phase" of treatment. After cessation of treatment, patients returned in 6 weeks (18 weeks from most recent treatment). If the patient remained stable at the 6-week follow up visit, treatment was held again, and the patient was followed every 12 weeks during the monitoring phase of the approach. A decline in vision, or a return in fluid or hemorrhage at subsequent visits would result in resuming treatment. Patients who failed an extension would be given another opportunity to have the interval between visits extended once they were stable for at least 3 consecutive visits. Patients were defined as having been successfully paused from treatment if they did not require treatment on 3 consecutive scheduled visits following treatment cessation (i.e., at least 12 + 6 + 12 weeks; or 30 weeks total). Follow up for patients successfully paused from anti-VEGF therapy therefore required a minimum of 30 weeks beyond their last injection.

Grading fluid status overtime

SD-OCT images were obtained and analyzed from all eligible patients enrolled in the study who underwent the TEP/M approach for at least 12 months. SD-OCT images were graded at timepoints 0, 1, 2, 3, 6, and 12 months after initiation of protocol for the presence of fluid by two

independent and masked graders (TPP and ZY); OCTs performed at time points closest to 6 and 12 months (i.e., within 4 weeks of the 6 time point and 6 weeks of the 12 month time point from treatment initiation) were included in this analysis. Each image was classified as having no fluid, subretinal fluid (SRF), intraretinal fluid (IRF), or both. Differences in image grading between the two blinded graders was reconciled and if an agreement could not be reached, a third investigator (AS) cast the tie-breaker vote. The location of fluid for each patient and at each time point was graphically represented in a table with different color cells representing no fluid, SRF, IRF or both. Patients were grouped based on frequency of treatment at 12 months to compare those who were able to successfully pause treatment with those who were unable to reach treatment pause.

Proteomics

Sample preparation

Samples from control patients, dry (non-neovascular) AMD patients, untreated nvAMD patients, and nvAMD patients (untreated and treated up to three times) from the TEP/M study who required monthly or q12 weeks (or longer) treatment at 1 year were used for proteomics studies. For each sample, ~10 µg proteins from pooled patient samples in 10µL of 8.0 M urea lysis buffer was reduced with 6 mM dithiothreitol (DTT) for 1h at 37 °C, and then alkylated with 12 mM iodoacetamide (IAA) for 45 minutes in the dark at room temperature. The proteins were then digested to peptides by Lys-C and trypsin. After the digestion, the peptides were acidified with 50% formic acid to pH <3, and then desalted on C18 stage-tip. Desalted peptides were then dried in a Speed Vacuum and then resuspended in 3% acetonitrile (ACN) with 0.1% HCOOH for future liquid chromatography mass spectrometry analysis (LC-MS/MS).

LC-MS/MS analysis using data independent acquisition (DIA)

1 ug peptides of each sample were spiked with iRT peptides and separated by ACN gradient on an in-house packed C18 column (0.75 μm I.D. x 26.5 cm length) on the Easy nLC 1200 UHPLC system (Thermo Scientific). Separated peptides were analyzed through Nanospray Flex™ Ion Source (Thermo Scientific) using Orbitrap Fusion™ Lumos™ Tribrid™ MS instrument (Thermo Scientific) by DIA as described previously (16).

Analysis of DIA data

The DIA data was analyzed using library free strategy. All the 9 DIA runs were performed using Spectronaut™(version 14.10, Biognosys, Schlieren, Switzerland) with directDIA™ analysis following the BGS factory search setting with uniprot_reviewed_human database (release 20,418 entries). The variable modifications on amino acid were set as oxidation(M) and acetylation (protein N-terminus), and the fixed modification on amino acid was set as carbamidomethylation (C). Cross run normalization was applied to normalize the DIA together with normalized on median.

Statistical Analyses for Proteomic Analyses

PCA analysis

Principal component analysis (PCA) was conducted using Scikit-learn (18), a machining learning module in python. The first 3 components (PCA1, PCA2, PCA3) were selected to simulate variances between 6 groups of patients (Q4 Untreated, Q4 Treated, Q12+ Untreated, Q12+Treated, Non-AMD Control, Dry AMD). Percentage of variance explained by the first 3 components were 54.73%, 42.78%, 2.16%, respectively.

Gene Ontology

For gene ontology analyses, DAVID was utilized to identify relevant biological processes enriched in the expressed proteins (19, 20). FDR =0.05 was chosen for the statistical significance.

Hierarchical clustering

Heatmap of protein expression level was generated using unsupervised hierarchical clustering with command of *scipy.cluster.hierarchy.linkage(method="complete", metric="euclidean"* from Scipy library (21) in python. The distance matrix used in the clustering was first constructed from z-score of protein expression across 6 groups of patients. Farthest Point algorithm (also known as *Complete*) was used to calculate Euclidean distance between 6 groups of patients. Visualization of heatmap was generated by Matplotlib library (22) in python.

Mice

Eight-week-old pathogen-free female C57BL/6 mice were obtained from Jackson Laboratory. *ApoB* mutant mice(17) and B6129 wild type control mice were obtained from Jackson Laboratories. The RD8 mutation was bred out of *ApoB* mutant mice and all mice were free of this mutation. All animals were treated in accordance with the Association for Research in Vision and Ophthalmology Statement for the Use of Animals in Ophthalmic and Vision Research and the guidelines of the Johns Hopkins University Animal Care and Use Committee.

Quantitative Real-Time PCR (RT-qPCR)

Total RNA was isolated from culture cells or retinas with PureLink™ RNA Mini Kit (Invitrogen #12183025), and cDNA was prepared with MuLV Reverse Transcriptase (Applied Biosystems). Quantitative real-time PCR was performed with Power SYBR Green PCR Master

Mix (Applied Biosystems) and MyiQ Real-Time PCR Detection System (Bio-Rad). Normalization was done using cyclophilin A. Total RNA was extracted using RNeasy Mini Kit (Ambion, Cat# 12183025), followed by reverse transcription to synthesize cDNA with QuantiTect Reverse Transcription Kit (Qiagen, Cat# 205311). Quantitative real-time PCR was performed using SYBR Green PCR Master Mix (Applied Biosystems, Cat# 25742). The following primers were used to examine VEGF and ApoB100 mRNA expression levels. Mouse VEGF forward primer, 5'-TTACTGCTGTACCTCCACC-3', and reverse primer, 5'-ACAGGACGGCTTGAAGATG-3'; mouse ApoB100 forward primer, 5'-AAGCACCTCCGAAAGTACGTG-3', and reverse primer, 5'-CTCCAGCTCTACCTTACAGTTGA-3'; mouse Cyclophilin A (internal control) forward primer, 5'-AGCATACAGGTCCTGGCATC-3', and reverse primer, 5'-TTCACCTTCCCAAAGACCAC-3'.

Laser CNV Model

Mice were anesthetized with a mixture of ketamine (100 mg/kg) and xylazine (5mg/kg). After pupils were dilated with tropicamide (Alcon Laboratories), lubricating hypromellose eye drops (Alcon Laboratories) were applied to the cornea. The fundus was viewed using a hand-held coverslip as a contact lens and laser photocoagulation was performed using the diode laser photocoagulator (IRIS Medical, Mountain View, CA) and the slit lamp delivery system. Four laser burn spots equidistant from the optic nerve using a wavelength of 532 nm, a power of 190 milliwatt, a duration of 100 ms, and a spot size of 75 μm were performed for each eye. In a subset of animals, 3 days after laser treatment, mice received a single intravitreal injection with aflibercept (200 ng/ul). Mice were sacrificed at day 7 and the eyes enucleated and fixed with 4% paraformaldehyde in PBS for 30 minutes at room temperature. Posterior eyecups

(RPE/choroid/sclera) were isolated and blocked in 5% bovine serum albumin (BSA) at 4 °C overnight and then stained with Alexa Fluor 594 conjugated Isolectin B4 (1:200, Invitrogen, CA) at 4 °C overnight. After staining, the eyecups were flat-mounted onto microscope slides with mounting medium. Lesions were excluded if visualization was obscured by overlying hemorrhage, or they were not round/discrete or if they merged into an adjacent lesion. Images were captured by Axioplan 2 imaging microscope (Carl Zeiss MicroImaging, Inc., Thornwood, NY). The area of the lesions was measured by Image J software. Representative images for selected time points from a minimum of three independent experiments are shown. Data from 4-6 animals were obtained at each time point.

Intraocular Injections

Intravitreal injections with aflibercept were performed with a with (PLI-100A) Pico-liter Microinjector (Warner Instruments, Harvard Bioscience) using pulled-glass micropipettes. Each micropipette was calibrated to deliver a 1-μL volume on depression of a foot switch. The mice were anesthetized with ketamine (100 mg/kg) and xylazine (5mg/kg) mixture and under a dissecting microscope after pupils were dilated with tropicamide (Alcon Laboratories). The sharpened tip of the micropipette was passed through the sclera just posterior to the limbus into the vitreous cavity and visualized through the dilated pupil. The foot switch was depressed, which caused fluid to penetrate into the vitreous space.

Supplemental Table 1

Supplemental Table 1. Eyes of TEP/M patients grouped by interval between treatments at 12 months.

Characteristic	q4	q6-8	q10-12	Weaned
Percent of Eyes at each interval - (no.)	17% (17)	31% (32)	21% (21)	31% (32)

Abbreviation: No., number; q4, requiring treatment every 4 weeks; q6-8, requiring treatment every 6-8 weeks; q10-12, requiring treatment every 10-12 weeks; TEP/M, treat and extend pause/monitor.

Supplemental Table 2

Supplemental Table 2. Recurrence rate at 24 and 36 months in eyes of TEP/M patients who were successfully weaned from anti-VEGF treatment from the prior year.

Characteristics	24 Months	36 Months
Number of eyes weaned off treatment from previous year	22	15
Eyes with recurrent activity in 12 months following weaning - % (no.)	27% (6)	13% (2)

Abbreviation: No., number; TEP/M, treat and extend pause/monitor.

Supplemental Table 3

Supplemental Table 3. Eyes of TEP/M patients successfully weaned from anti-VEGF treatment at any timepoint in the first 36 months of their treatment course.

**Characteristics**	
Total number of eyes	102
Median length of follow-up in months (IQR)	28.5 (18.0 - 43.5)
Range of follow-up in months	12 – 72
Percent of eyes that were successfully weaned at any point during follow-up - (no.)	43% (44)
Mean length of pause without treatment for all patients who reached the monitoring phase (months) ± SEM	18.0 ± 1.8

Abbreviation: No., number. IQR, interquartile range; SEM, standard error of the mean; TEP/M, treat and extend pause/monitor.

Supplemental Table 4

Supplemental Table 4. Comparison of TEP/M eyes requiring "maintenance therapy" every 2-3 months with eyes of patients successfully weaned from treatment at 12 months.

**Characteristic**		**q8-12**	**Weaned**	***P***
Number of Eyes		35	32	
Change in Vision (ETDRS letters)		+1 ± 3	+4 ± 3	0.401
Letters Gained - % (no.)	5 or more letters	**34% (12)**	**53% (17)**	**0.007**
	10 or more letters	26% (9)	31% (10)	0.434
	15 or more letters	20% (7)	16% (5)	0.462
Letters Lost - % (no.)	5 or more letters	17% (6)	19% (6)	0.713
	10 or more letters	14% (5)	9% (3)	0.268
	15 or more letters	14% (5)	6% (2)	0.059
Change in CST (μm)		-80.6 ± 18.6	-70.4 ± 13.3	0.861
Mean # of Treatments		**7.8 ± 0.3**	**6.0 ± 0.3**	**<0.0001**

Abbreviations: No., number; ETDRS, Early Treatment Diabetic Retinopathy Study letter score. CST, central subfield thickness; q8-12, requiring treatment every 8 -12 weeks; TEP/M, treat and extend pause/monitor. Values displayed as mean ± standard error of the mean. Statistical analysis was performed using Chi-square and Mann-Whitney test. Data in bold are statistically significant.

Supplemental Table 5

Supplemental Table 5. Recovery of vision after re-treatment with anti-VEGF in patients who were weaned from therapy but were later diagnosed with new CNV activity.

**Characteristic**	
Number of Eyes with recurrent activity	8
Vision at presentation (ETDRS letters)	61 ± 5
Vision at time of weaning (ETDRS letters)	67 ± 4
Vision at time of diagnosis of recurrence (ETDRS letters)	61 ± 5
Change in vision between time of weaning and time of diagnosis of recurrent activity (ETDRS letters)	-6 ± 2
Vision at first visit after retreatment (ETDRS letters)	64 ± 5
Recovery of vision between time of diagnosis of recurrence and first visit after retreatment (ETDRS letters)	+3 ± 2

Abbreviations: ETDRS, Early Treatment Diabetic Retinopathy Study letter score. Values displayed as mean ± standard error of the mean.

Supplemental Table 6

Supplemental Table 6. Summary of adverse events following treatment initiation at 24 and 36 months for TEP/M patients.

		24 months				36 months			
**Treatment Interval**		**q4-6**	**q8-12**	**Weaned**	***P***	**q4-6**	**q8-12**	**Weaned**	***P***
Number of Eyes		25	22	23		16	9	14	
Adverse Events	Endophthalmitis	0	0	0	-	0	0	0	-
	Retinal Tear/Detachment	0	0	0	-	0	0	0	-
	Vitreous Hemorrhage	0	1	0	0.331	1	1	0	0.482
	RPE Tear	1	0	0	0.401	1	0	0	0.478
	SRH	4	1	0	0.084	4	1	0	0.122
	SR fibrosis	2	1	2	0.846	2	1	2	0.974

Abbreviations: SRH, subretinal hemorrhage; RPE, retinal pigment epithelium; SR fibrosis, subretinal fibrosis. q4-6, every 4-6 weeks; q8-12, every 8 to 12 weeks; TEP/M, treat and extend pause/monitor. Adverse events were recorded over the initial three years of the study for patients with a minimum of 24 months of follow-up up to a maximum of 36 months of follow-up. Statistical analysis was performed using 3-sample test for equality of proportions.

Supplemental Table 7

Supplemental Table 7. Baseline characteristics of patients used in VEGF ELISA.

**Characteristic**	**Pre-treatment q4 (n=6)**	**Pre-treatment q6-8 (n=5)**	**Pre-treatment q10-12 (n=3)**	**Pre-treatment Weaned (n=9)**	**Post-treatment q4 (n=4)**	**Post-treatment q6-8 (n=1)**	**Post-treatment q10-12 (n=4)**	**Post-treatment Weaned (n=3)**	**Control (n=23)**	***P***
Mean Age (yr)	78.3 ± 1.5	83.4 ± 2.5	82.3 ± 0.9	83.0 ± 2.2	75.0 ± 0.0	75.0*	78.0 ± 3.5	88.3 ± 2.0	71.2 ± 1.9	**<0.001**
Female % (#)	83% (5)	60% (3)	100% (3)	78% (7)	50% (2)	100% (1)	50% (2)	100% (3)	43% (10)	0.782
Pseudophakic % (#)	83% (5)	100% (5)	67% (2)	89% (8)	100% (4)	100% (1)	0% (0)	100% (3)	39% (9)	0.183

Abbreviations: n, sample size; q4, received treatment every 4 weeks; q6-8, received treatment every 6-8 weeks; q10-12, received treatment every 10-12 weeks; VEGF, vascular endothelial growth factor; ELISA, enzyme-linked immunoassay; and yr, years. Values displayed as mean ± standard error of the mean. *Unable to obtain standard error of the mean with a sample size of 1. Statistical analysis was performed using ANOVA and Fisher's exact test. Data in bold are statistically significant.

Supplemental Table 8

Supplemental Table 8. Baseline characteristics of patients used in the proteomic screen.

Characteristic	Untreated q4 (n=3)	Untreated q12+ (n=7)	Treated q4 (n=6)	Treated q12+ (n=5)	nnvAMD (n=18)	Untreated nvAMD 1 (n=9)	Untreated nvAMD 2 (n=9)	Control 1 (n=12)	Control 2 (n=12)	*P*
Mean Age (yr)	81.7 ± 2.3	83.7 ± 2.4	75.0 ± 2.4	82.6 ± 5.0	74.8 ± 2.6	80.2 ± 3.7	80.7 ± 2.1	71.1 ± 2.8	70.5 ± 2.5	**0.008**
Female % (#)	67% (2)	86% (6)	50% (3)	60% (3)	61% (11)	56% (5)	56% (5)	42% (5)	42% (5)	0.681
Pseudophakic % (#)	67% (2)	86% (6)	100% (6)	80% (4)	0% (0)	56% (5)	89% (8)	50% (6)	25% (3)	**<0.001**

Abbreviations: n, sample size; q4, received treatment every 4 weeks; q12+, received treatment every 12 or more weeks; nnvAMD, non-neovascular Age-related Macular Degeneration; nvAMD, neovascular Age-related Macular Degeneration; and yr, years. Values displayed as mean ± standard error of the mean. Statistical analysis was performed using ANOVA and Fisher’s exact test. Data in bold are statistically significant.

Supplemental Table 9. Proteins identified in Proteomic Analyses which had sequences that overlap with current anti-VEGF therapies.

Cohort	Protein Accessions	Genes	Protein
Untreated q12+ vs. q4 (n=34)	P01764	IGHV3-23	Immunoglobulin heavy variable 3-23
	P01834	IGKC	Immunoglobulin kappa constant
	P0DOX7	-	Immunoglobulin kappa light chain
	P01624	IGKV3-15	Immunoglobulin kappa variable 3-15
	A0A0C4DH31	IGHV1-18	Immunoglobulin heavy variable 1-18
	A0A0B4J1V2	IGHV2-26	Immunoglobulin heavy variable 2-26
	P01706	IGLV2-11	Immunoglobulin lambda variable 2-11
	P0DOX3	-	Immunoglobulin delta heavy chain
	P01700	IGLV1-47	Immunoglobulin lambda variable 1-47
	A0A0J9YX35	IGHV3-64D	Immunoglobulin heavy variable 3-64D
	A0M8Q6	IGLC7	Immunoglobulin lambda constant 7
	A0A087WSZ0	IGKV1D-8	Immunoglobulin kappa variable 1D-8
	P15814	IGLL1	Immunoglobulin lambda-like polypeptide 1
	A0A0B4J1V6	IGHV3-73	Immunoglobulin heavy variable 3-73
	A0A0B4J1V0	IGHV3-15	Immunoglobulin heavy variable 3-15
	P01619	IGKV3-20	Immunoglobulin kappa variable 3-20
	A0A0C4DH29	IGHV1-3	Immunoglobulin heavy variable 1-3
	P01591	JCHAIN	Immunoglobulin J chain
	P01704	IGLV2-14	Immunoglobulin lambda variable 2-14
	P01599	IGKV1-17	Immunoglobulin kappa variable 1-17
	A0A075B6K5	IGLV3-9	Immunoglobulin lambda variable 3-9
	P06310	IGKV2-30	Immunoglobulin kappa variable 2-30
	P17948	FLT1	Vascular endothelial growth factor receptor 1
	A0A087WW87	IGKV2-40	Immunoglobulin kappa variable 2-40
	A0A0C4DH43	IGHV2-70D	Immunoglobulin heavy variable 2-70D
	A0A0C4DH25	IGKV3D-20	Immunoglobulin kappa variable 3D-20
	A0A0B4J1X8	IGHV3-43	Immunoglobulin heavy variable 3-43
	P01602	IGKV1-5	Immunoglobulin kappa variable 1-5
	P01593	IGKV1D-33	Immunoglobulin kappa variable 1D-33
	P01709	IGLV2-8	Immunoglobulin lambda variable 2-8
	P04211	IGLV7-43	Immunoglobulin lambda variable 7-43
	P01703	IGLV1-40	Immunoglobulin lambda variable 1-40
	P01715	IGLV3-1	Immunoglobulin lambda variable 3-1
	P06312	IGKV4-1	Immunoglobulin kappa variable 4-1
Treated q12+ vs. q4 (n=15)	A0A0A0MT36	IGKV6D-21	Immunoglobulin kappa variable 6D-21
	A0A075B6I0	IGLV8-61	Immunoglobulin lambda variable 8-61
	P01766	IGHV3-13	Immunoglobulin heavy variable 3-13
	A0A0A0MT89	IGKJ1	Immunoglobulin kappa joining 1
	A0A0B4J1V1	IGHV3-21	Immunoglobulin heavy variable 3-21
	A0A0C4DH67	IGKV1-8	Immunoglobulin kappa variable 1-8
	P0DP01	IGHV1-8	Immunoglobulin heavy variable 1-8
	O75015	FCGR3B	Low affinity immunoglobulin gamma Fc region receptor III-B
	P0DOX5	-	Immunoglobulin gamma-1 heavy chain
	P01859	IGHG2	Immunoglobulin heavy constant gamma 2
	A0A075B6S2	IGKV2D-29	Immunoglobulin kappa variable 2D-29
	A0A075B6Q5	IGHV3-64	Immunoglobulin heavy variable 3-64
	P01782	IGHV3-9	Immunoglobulin heavy variable 3-9
	A0A0C4DH68	IGKV2-24	Immunoglobulin kappa variable 2-24
	Q9Y279	VSIG4	V-set and immunoglobulin domain-containing protein 4
Untreated q12+ vs. q4 + Treated q12+ vs. q4 (n=9)	P35968	KDR	Vascular endothelial growth factor receptor 2
	P01871	IGHM	Immunoglobulin heavy constant mu
	P01861	IGHG4	Immunoglobulin heavy constant gamma 4
	P08637	FCGR3A	Low affinity immunoglobulin gamma Fc region receptor III-A
	A0A075B6K0	IGLV3-16	Immunoglobulin lambda variable 3-16
	A0A075B6I9	IGLV7-46	Immunoglobulin lambda variable 7-46
	P01705	IGLV2-23	Immunoglobulin lambda variable 2-23
	P0DOX2	-	Immunoglobulin alpha-2 heavy chain
	A0A075B6R2	IGHV4-4	Immunoglobulin heavy variable 4-4

Supplemental Table 10. Proteins identified in Proteomics Analyses that were "highly variable".

Cohort	Protein Accessions	Genes	Protein
Untreated q12+ vs. q4 (n=14)	O95897	OLFM2	Noelin-2
	P02144	MB	Myoglobin
	P02533	KRT14	Keratin, type I cytoskeletal 14
	P02745	C1QA	Complement C1q subcomponent subunit A
	P07996	THBS1	Thrombospondin-1
	P30838	ALDH3A1	Aldehyde dehydrogenase, dimeric NADP-preferring
	Q06033	ITIH3	Inter-alpha-trypsin inhibitor heavy chain H3
	Q7Z7M0	MEGF8	Multiple epidermal growth factor-like domains protein 8
	Q92859	NEO1	Neogenin
	P02538	KRT6A	Keratin, type II cytoskeletal 6A
	P13645	KRT10	Keratin, type I cytoskeletal 10
	P15121	AKR1B1	Aldose reductase
	P30041	PRDX6	Peroxiredoxin-6
	P29401	TKT	Transketolase
Treated q12+ vs. q4 (n=9)	P27169	PON1	Serum paraoxonase/arylesterase 1
	Q08554	DSC1	Desmocollin-1
	Q9BQT9	CLSTN3	Calsyntenin-3
	A0A140G945	CRYAA2	Alpha-crystallin A2 chain
	P02743	APCS	Serum amyloid P-component
	P16152	CBR1	Carbonyl reductase [NADPH] 1
	P26998	CRYBB3	Beta-crystallin B3
	Q9BZV3	IMPG2	Interphotoreceptor matrix proteoglycan 2
	P53672	CRYBA2	Beta-crystallin A2
Untreated q12+ vs. q4 + Treated q12+ vs. q4 (n=8)	P04264	KRT1	Keratin, type II cytoskeletal 1
	P11226	MBL2	Mannose-binding protein C
	P35908	KRT2	Keratin, type II cytoskeletal 2 epidermal
	P53674	CRYBB1	Beta-crystallin B1
	P05408	SCG5	Neuroendocrine protein 7B2
	P26447	S100A4	Protein S100-A4
	P35527	KRT9	Keratin, type I cytoskeletal 9
	P69891	HBG1	Hemoglobin subunit gamma-1
Non-Categorized Highly Variable nvAMD Proteins (n=20)	O76061	STC2	Stanniocalcin-2
	P00742	F10	Coagulation factor X
	P02654	APOC1	Apolipoprotein C-I
	P02656	APOC3	Apolipoprotein C-III
	P02766	TTR	Transthyretin
	P02795	MT2A	Metallothionein-2
	P03950	ANG	Angiogenin
	P03973	SLPI	Antileukoproteinase
	P04908	HIST1H2AB	Histone H2A type 1-B/E
	P15291	B4GALT1	Beta-1,4-galactosyltransferase 1
	P31025	LCN1	Lipocalin-1
	P42857	NSG1	Neuronal vesicle trafficking-associated protein 1
	P80188	LCN2	Neutrophil gelatinase-associated lipocalin
	Q08380	LGALS3BP	Galectin-3-binding protein
	Q14114	LRP8	Low-density lipoprotein receptor-related protein 8
	Q14520	HABP2	Hyaluronan-binding protein 2
	Q7L804	RAB11FIP2	Rab11 family-interacting protein 2
	Q8IUX7	AEBP1	Adipocyte enhancer-binding protein 1
	Q92520	FAM3C	Protein FAM3C
	Q96HF1	SFRP2	Secreted frizzled-related protein 2

"Highly variable" proteins were those proteins identified in the Proteomics comparison of NVAMD patients that were arbitrarily divided into two group: NVAMD1 and NVAMD2. Proteins were designated as "highly variable" if they were increased or decreased two-fold or more in this comparison.

Supplemental Table 11. Proteins identified in Proteomics Analyses comparing untreated q12+ vs q4.

Genes	Protein	Untreated [q12+] / [q4] Fold Induction	Families	Overlap in other groups
HBB	Hemoglobin subunit beta	294.5912433	blood coagulation	T, DW
HBD	Hemoglobin subunit delta	133.9940856	blood coagulation	DW
FGA	Fibrinogen alpha chain	2.515216354	blood coagulation, innate immune response	T
FGG	Fibrinogen gamma chain	2.44139284	Blood coagulation	T
PRDX2	Peroxiredoxin-2	40.31231115	response to oxidative stress	DW
CA3	Carbonic anhydrase 3	12.6337525	response to oxidative stress	DW
RS1	Retinoschisin	5.352000599	visual perception	
KERA	Keratocan	3.034881465	visual perception	
UBB	Polyubiquitin-B	5.148367901	innate immune response	DW
CD14	Monocyte differentiation antigen CD14	3.637119709	innate immune response, Inflammatory response	
CHGA	Chromogranin-A	2.143352597	innate immune response	
TGFBI	Transforming growth factor-beta-induced protein ig-h3	3.191129815	angiogenesis, visual perception	
FGFR1	Fibroblast growth factor receptor 1	2.32459108	Angiogenesis	
HRG	Histidine-rich glycoprotein	2.009087578	angiogenesis	
PARK7	Protein/nucleic acid deglycase DJ-1	2.825504266	inflammatory response	DW
ACTN4	Alpha-actinin-4	2.769172538	response to hypoxia	
CTSC	Dipeptidyl peptidase 1	2.329260204	aging, immune response	
HBA1	Hemoglobin subunit alpha	338.3618802		T, DW
BFSP1	Filensin	324.6129602		
CA1	Carbonic anhydrase 1	279.0881896		T, DW
UCHL1	Ubiquitin carboxyl-terminal hydrolase isozyme L1	15.46238334		DW
CA2	Carbonic anhydrase 2	11.8927982		DW
PRDX1	Peroxiredoxin-1	11.5138349		DW
LTBP1	Latent-transforming growth factor beta-binding protein 1	8.552371289		
GPR37	Prosaposin receptor GPR37	7.333329359		
CFHR4	Complement factor H-related protein 4	6.394024547		DW
S100A6	Protein S100-A6	6.364036426		
QPCT	Glutaminyl-peptide cyclotransferase	6.212174994		
SERPINB6	Serpin B6	5.844286537		DW
KRT13	Keratin, type I cytoskeletal 13	5.68192904		DW
SHBG	Sex hormone-binding globulin	4.635185611		
NEB	Nebulin	4.362882999		T
GALE	UDP-glucose 4-epimerase	4.349337472		
TYRP1	5,6-dihydroxyindole-2-carboxylic acid oxidase	4.348768788		
COL9A3	Collagen alpha-3(IX) chain	4.331643822		
CFL1	Cofilin-1	4.196064163		
ERN1	Serine/threonine-protein kinase/endoribonuclease IRE1	3.793809256		DW
HPR	Haptoglobin-related protein	3.742294514		DW
OMD	Osteomodulin	3.730660593		
YWHAE	14-3-3 protein epsilon	3.727339924		DW
NME1	Nucleoside diphosphate kinase A	3.724068254		
LYNX1	Ly-6/neurotoxin-like protein 1	3.659804308		
ADAMTS1	A disintegrin and metalloproteinase with thrombospondin motifs 1	3.587495102		T
VGF	Neurosecretory protein VGF	3.407534525		
SERPINA6	Corticosteroid-binding globulin	3.257955543		DW
YIPF3	Protein YIPF3	3.078472751		
ITM2B	Integral membrane protein 2B	2.921995474		
PEBP4	Phosphatidylethanolamine-binding protein 4	2.887451256		T
SEZ6	Seizure protein 6 homolog	2.830353002		
A2M	Alpha-2-macroglobulin	2.72326802		
DKK3	Dickkopf-related protein 3	2.679453271		
AGA	N(4)-(beta-N-acetylglucosaminyl)-L-asparaginase	2.663244962		
HSPA8	Heat shock cognate 71 kDa protein	2.635605532		T
ICOSLG	ICOS ligand	2.44353239		
APOH	Beta-2-glycoprotein 1	2.362134509		
MINPP1	Multiple inositol polyphosphate phosphatase 1	2.298580776		
ESD	S-formylglutathione hydrolase	2.272286671		
OGN	Mimecan	2.243984496		DW
PRKCSH	Glucosidase 2 subunit beta	2.20073281		
YWHAQ	14-3-3 protein theta	2.190339748		
NRXN2	Neurexin-2	2.102067281		
CYTL1	Cytokine-like protein 1	2.080244258		
HEXA	Beta-hexosaminidase subunit alpha	2.052336483		
PPIA	Peptidyl-prolyl cis-trans isomerase A	2.050585783		DW
ALDOA	Fructose-bisphosphate aldolase A	2.012219378		T, DW

Supplemental Table 11. Continued…

Genes	Protein	Untreated [q12+] / [q4] Fold Induction	Families	Overlap in other groups
CRYAB	Alpha-crystallin B chain	0.163975792	aging, response to hypoxia	DW
SERPING1	Plasma protease C1 inhibitor	0.333826255	aging, innate immune response	
TIMP2	Metalloproteinase inhibitor 2	0.461901398	aging	
FN1	Fibronectin	0.185015609	angiogenesis, response to wounding	
FABP5	Fatty acid-binding protein 5	0.248945277	response to wounding	DW
CHI3L1	Chitinase-3-like protein 1	0.254186936	inflammatory response	
MIF	Macrophage migration inhibitory factor	0.285407022	innate immune response, Inflammatory response	DW
C4A	Complement C4-A	0.373871058	innate immune response, Inflammatory response	
S100B	Protein S100-B	0.457245901	innate immune response	DW
COL1A1	Collagen alpha-1(I) chain	0.313111308	blood coagulation, visual perception	
COL1A2	Collagen alpha-2(I) chain	0.316924771	blood coagulation	
PLG	Plasminogen	0.407741011	blood coagulation	
PROZ	Vitamin K-dependent protein Z	0.423337066	blood coagulation	T
SERPINA1	Alpha-1-antitrypsin	0.496211675	blood coagulation	
CRYBA4	Beta-crystallin A4	0.357924479	visual perception	DW
RBP4	Retinol-binding protein 4	0.386044119	visual perception	
CRYBA1	Beta-crystallin A3	0.448229078	visual perception	DW
PRG4	Proteoglycan 4	0.435270018	immune response	
PKM	Pyruvate kinase PKM	0.446404568	response to hypoxia	DW
PON3	Serum paraoxonase/lactonase 3	7.80919E-05		
SPRR2E	Small proline-rich protein 2E	0.028155793		
ANXA2P2	Putative annexin A2-like protein	0.072406169		DW
SEMA3A	Semaphorin-3A	0.083599748		DW
FCN3	Ficolin-3	0.133115105		DW
CD163	Scavenger receptor cysteine-rich type 1 protein M130	0.139077514		T
KRT5	Keratin, type II cytoskeletal 5	0.172802231		T, DW
CLN5	Ceroid-lipofuscinosis neuronal protein 5	0.181945084		
EEF1A1	Elongation factor 1-alpha 1	0.187204338		
PLTP	Phospholipid transfer protein	0.221588788		
ACTG1	Actin, cytoplasmic 2	0.233803389		DW
HSPB1	Heat shock protein beta-1	0.250143493		DW
NCAM1	Neural cell adhesion molecule 1	0.27083065		
CLUL1	Clusterin-like protein 1	0.271490215		DW
PCSK2	Neuroendocrine convertase 2	0.275888468		
SEMA3F	Semaphorin-3F	0.286041058		
CLSTN2	Calsyntenin-2	0.302221064		
WIF1	Wnt inhibitory factor 1	0.315781286		
WFDC1	WAP four-disulfide core domain protein 1	0.319196958		
CADM1	Cell adhesion molecule 1	0.339567104		
APOB	Apolipoprotein B-100	0.351268533		T, DW
HSP90AB1	Heat shock protein HSP 90-beta	0.355196334		T, DW
KRT86	Keratin, type II cuticular Hb6	0.356898821		
GRN	Progranulin	0.366381115		
CDON	Cell adhesion molecule-related/down-regulated by oncogenes	0.379931695		T
SPARC	SPARC	0.392199239		
CTSZ	Cathepsin Z	0.39284597		T
VIM	Vimentin	0.403779431		DW
OAF	Out at first protein homolog	0.406433181		
FSTL3	Follistatin-related protein 3	0.406715597		
FCGBP	IgGFc-binding protein	0.417994546		T
EFEMP2	EGF-containing fibulin-like extracellular matrix protein 2	0.420191938		
GAS6	Growth arrest-specific protein 6	0.420795231		
SHISA5	Protein shisa-5	0.423531653		
IGFBP3	Insulin-like growth factor-binding protein 3	0.43147223		
HSPA5	Endoplasmic reticulum chaperone BiP	0.432265466		
SORD	Sorbitol dehydrogenase	0.43334378		DW
CFHR5	Complement factor H-related protein 5	0.458636856		
COMP	Cartilage oligomeric matrix protein	0.462606424		
BGN	Biglycan	0.472085944		DW
CDH13	Cadherin-13	0.47672027		
ORM2	Alpha-1-acid glycoprotein 2	0.480971172		
SEMA4B	Semaphorin-4B	0.482576005		T, DW
FSTL4	Follistatin-related protein 4	0.489451605		DW
PDIA3	Protein disulfide-isomerase A3	0.494677659		
CHRDL1	Chordin-like protein 1	0.496701195		

Abbreviations: T, treated; q12+ vs. q4; and DW, dry (nnv) vs. wet (nv) AMD.

Supplemental Table 12. Proteins identified in Proteomics Analyses comparing treated q12+ vs q4.

Genes	Protein	Treated [q12+] / [q4] Fold Induction	Families	Overlap in other groups
HBB	Hemoglobin subunit beta	9.741177638	blood coagulation	U, DW
VWF	von Willebrand factor	2.316170198	blood coagulation, response to wounding	
F13B	Coagulation factor XIII B chain	2.071380744	blood coagulation	
PPBP	Platelet basic protein	5.938169813	immune response, inflammatory response	
LRP1	Prolow-density lipoprotein receptor-related protein 1	2.256691398	aging	
REG3A	Regenerating islet-derived protein 3-alpha	24.49209647		
APOB	Apolipoprotein B-100	12.5123028		U, DW
LPA	Apolipoprotein(a)	7.348682752		
LYPD3	Ly6/PLAUR domain-containing protein 3	6.46264229		
HBA1	Hemoglobin subunit alpha	6.265305549		U, DW
HSP90AB1	Heat shock protein HSP 90-beta	4.567443665		U, DW
CREG1	Protein CREG1	3.69341753		
CA1	Carbonic anhydrase 1	3.679594987		U, DW
SAA2	Serum amyloid A-2 protein	3.592598983		
IGFALS	Insulin-like growth factor-binding protein complex acid labile subunit	3.033746866		DW
ALDOA	Fructose-bisphosphate aldolase A	2.845715536		U, DW
NEB	Nebulin	2.579059473		U
ADAMTS1	A disintegrin and metalloproteinase with thrombospondin motifs 1	2.556413438		U
PTPRG	Receptor-type tyrosine-protein phosphatase gamma	2.411555991		
SERPINE3	Serpin E3	2.270504964		
DPP7	Dipeptidyl peptidase 2	2.266958417		
DNASE2	Deoxyribonuclease-2-alpha	2.241166621		
SEMA4B	Semaphorin-4B	2.225358877		U, DW
XYLT1	Xylosyltransferase 1	2.145490963		
LSAMP	Limbic system-associated membrane protein	2.128989213		
CDHR1	Cadherin-related family member 1	2.11348854		
CDON	Cell adhesion molecule-related/down-regulated by oncogenes	2.065481663		U
TFF3	Trefoil factor 3	2.061443766		
CARTPT	Cocaine- and amphetamine-regulated transcript protein	2.036529656		DW
PEBP4	Phosphatidylethanolamine-binding protein 4	2.029142013		U
C4BPB	C4b-binding protein beta chain	0.263806239	blood coagulation, innate immune response	DW
PROZ	Vitamin K-dependent protein Z	0.311111825	Blood coagulation	U
FGG	Fibrinogen gamma chain	0.414934313	Blood coagulation	U
FGA	Fibrinogen alpha chain	0.445736521	blood coagulation, innate immune response	U
F11	Coagulation factor XI	0.494791772	blood coagulation	DW
GSS	Glutathione synthetase	0.372327886	aging, response to oxidative stress	
APOD	Apolipoprotein D	0.442711612	aging, angiogenesis	
LYVE1	Lymphatic vessel endothelial hyaluronic acid receptor 1	0.379804515	response to wounding	
BFSP2	Phakinin	0.418796856	visual perception	DW
CRYBB2	Beta-crystallin B2	0.493134509	visual perception	DW
C3	Complement C3	0.459520524	immune response, inflammatory response	
DSG1	Desmoglein-1	0.172452156		
PCSK9	Proprotein convertase subtilisin/kexin type 9	0.180707873		
NOTCH2NLB	Notch homolog 2 N-terminal-like protein B	0.237657357		DW
KRT5	Keratin, type II cytoskeletal 5	0.365592975		U, DW
HSPA8	Heat shock cognate 71 kDa protein	0.392311831		U
C18orf63	Uncharacterized protein C18orf63	0.410397655		DW
HRNR	Hornerin	0.435647542		
RARRES1	Retinoic acid receptor responder protein 1	0.447729551		DW
CTSZ	Cathepsin Z	0.451195853		U
CPN2	Carboxypeptidase N subunit 2	0.458456923		
MST1	Hepatocyte growth factor-like protein	0.461035723		
ITIH2	Inter-alpha-trypsin inhibitor heavy chain H2	0.463211816		
FCGBP	IgGFc-binding protein	0.465329804		U
CTSH	Pro-cathepsin H	0.4686896		
NID1	Nidogen-1	0.478679154		
BLVRB	Flavin reductase (NADPH)	0.48032029		DW
SAA4	Serum amyloid A-4 protein	0.48042659		
CFHR2	Complement factor H-related protein 2	0.484750612		
MXRA5	Matrix-remodeling-associated protein 5	0.485991029		
CD163	Scavenger receptor cysteine-rich type 1 protein M130	0.493570136		U

Abbreviations: U, untreated q12+ vs. q4; and DW, dry vs. wet AMD.

Supplemental Table 13. Proteins identified in Proteomics Analyses comparing nnvAMD vs nvAMD patients.

Genes	Protein	[nnvAMD] / [nvAMD] Fold Induction	Families	Overlap in other groups
CRYGC	Gamma-crystallin C	61.08317488	visual perception	
CRYBA4	Beta-crystallin A4	52.012763	visual perception	U
CRYBA1	Beta-crystallin A3	46.68715798	visual perception	U
CRYBB2	Beta-crystallin B2	45.73386584	visual perception	T
CRYGD	Gamma-crystallin D	26.00092606	visual perception	
BFSP2	Phakinin	11.02043055	visual perception	T
CRYAB	Alpha-crystallin B chain	45.43108521	aging, response to hypoxia	U
SOD1	Superoxide dismutase [Cu-Zn]	2.754327935	aging	
S100B	Protein S100-B	13.70747112	innate immune response	U
MIF	Macrophage migration inhibitory factor	4.166874242	innate immune response, inflammatory response	U
UBB	Polyubiquitin-B	3.041668835	innate immune response	U
PARK7	Protein/nucleic acid deglycase DJ-1	7.132657804	inflammatory response	U
PRDX5	Peroxiredoxin-5, mitochondrial	2.864838425	inflammatory response, response to oxidative stress	
HBB	Hemoglobin subunit beta	21.05436769	blood coagulation	U, T
HBD	Hemoglobin subunit delta	8.367414187	blood coagulation	U
FABP5	Fatty acid-binding protein 5	5.923981916	response to wounding	U
PRDX2	Peroxiredoxin-2	4.616143292	response to oxidative stress	U
CA3	Carbonic anhydrase 3	3.128389633	response to oxidative stress	U
PKM	Pyruvate kinase PKM	3.875686939	response to hypoxia	U
LDHA	L-lactate dehydrogenase A chain	2.290805187	response to hypoxia	
GPI	Glucose-6-phosphate isomerase	2.137825433	angiogenesis	
VEGFA	Vascular endothelial growth factor A	2.006347773	angiogenesis, response to hypoxia	
CADM4	Cell adhesion molecule 4	1079.311912		
MTPN	Myotrophin	80.90222662		
UCHL1	Ubiquitin carboxyl-terminal hydrolase isozyme L1	68.27128776		U
HSPB1	Heat shock protein beta-1	20.7628404		U
HBA1	Hemoglobin subunit alpha	19.08833539		U, T
CRYGS	Gamma-crystallin S	15.57552607		
CA1	Carbonic anhydrase 1	14.67650386		U, T
ACTG1	Actin, cytoplasmic 2	14.50841714		U
PGK1	Phosphoglycerate kinase 1	10.83028208		
PEA15	Astrocytic phosphoprotein PEA-15	8.798078837		
SERPINB6	Serpin B6	8.324658874		U
GLO1	Lactoylglutathione lyase	8.083083466		
ALDOC	Fructose-bisphosphate aldolase C	7.694774407		
BLVRB	Flavin reductase (NADPH)	6.432180974		T
PPIA	Peptidyl-prolyl cis-trans isomerase A	6.224882713		U
CA2	Carbonic anhydrase 2	5.974602711		U
HSP90AB1	Heat shock protein HSP 90-beta	5.77152696		U, T
PEBP1	Phosphatidylethanolamine-binding protein 1	5.05793535		
VIM	Vimentin	5.0172362		U
CDA	Cytidine deaminase	4.922377169		
GDI1	Rab GDP dissociation inhibitor alpha	4.576977321		
COTL1	Coactosin-like protein	4.358400956		
PTMA	Prothymosin alpha	4.32518083		
SORD	Sorbitol dehydrogenase	4.06813868		U
CTSF	Cathepsin F	3.560278334		
ALDOA	Fructose-bisphosphate aldolase A	3.424663922		U, T
FABP3	Fatty acid-binding protein, heart	3.201090179		
YWHAE	14-3-3 protein epsilon	2.985849473		U
MDH1	Malate dehydrogenase, cytoplasmic	2.94065533		
TPI1	Triosephosphate isomerase	2.93747156		
CNDP2	Cytosolic non-specific dipeptidase	2.834570565		
PRDX1	Peroxiredoxin-1	2.750944724		U
AGRN	Agrin	2.612258344		
PLA2G2A	Phospholipase A2, membrane associated	2.388076841		
TTN	Titin	2.348953176		
TUBA1A	Tubulin alpha-1A chain	2.148832413		
DBI	Acyl-CoA-binding protein	2.133446259		
GPNMB	Transmembrane glycoprotein NMB	2.130059261		
FAH	Fumarylacetoacetase	2.12041099		
GAA	Lysosomal alpha-glucosidase	2.010706804		

Supplemental Table 13. Continued...

Genes	Protein	[nnvAMD] / [nvAMD] Fold Induction	Families	Overlap in other groups
NQO1	NAD(P)H dehydrogenase [quinone] 1	0.228294263	aging	
F11	Coagulation factor XI	0.229890141	blood coagulation	T
SERPINA10	Protein Z-dependent protease inhibitor	0.2728352	blood coagulation	
PROC	Vitamin K-dependent protein C	0.467077313	blood coagulation	
C4BPB	C4b-binding protein beta chain	0.47378127	blood coagulation, innate immune response	T
CD59	CD59 glycoprotein	0.477809186	blood coagulation	
CFI	Complement factor I	0.274247036	innate immune response	
APOL1	Apolipoprotein L1	0.44391838	innate immune response	
CSF1	Macrophage colony-stimulating factor 1	0.489228781	innate immune response, inflammatory response	
CHIT1	Chitotriosidase-1	0.40655903	immune response	
C8B	Complement component C8 beta chain	0.477202815	immune response	
ADIPOQ	Adiponectin	0.437976262	response to hypoxia	
SPTB	Spectrin beta chain, erythrocytic	0.000598752		
CFHR4	Complement factor H-related protein 4	0.003871227		U
FCN3	Ficolin-3	0.052762249		U
C18orf63	Uncharacterized protein C18orf63	0.070034178		T
KRT13	Keratin, type I cytoskeletal 13	0.116182893		U
APOB	Apolipoprotein B-100	0.16153718		U, T
ANK1	Ankyrin-1	0.185618017		
-	-	0.28871739		
LCAT	Phosphatidylcholine-sterol acyltransferase	0.289392178		
KRT5	Keratin, type II cytoskeletal 5	0.300217468		U, T
NOTCH2NLB	Notch homolog 2 N-terminal-like protein B	0.309663806		T
SLC3A2	4F2 cell-surface antigen heavy chain	0.33564667		
BGN	Biglycan	0.337024234		U
HPR	Haptoglobin-related protein	0.343944767		U
ERN1	Serine/threonine-protein kinase/endoribonuclease IRE1	0.349400822		U
ANXA2P2	Putative annexin A2-like protein	0.351552024		U
ALB	Serum albumin	0.373444253		
SPON1	Spondin-1	0.383488632		
CARTPT	Cocaine- and amphetamine-regulated transcript protein	0.387515798		T
TPP1	Tripeptidyl-peptidase 1	0.395130977		
CPN1	Carboxypeptidase N catalytic chain	0.403470064		
CDH5	Cadherin-5	0.417058529		
THBS4	Thrombospondin-4	0.426651703		
PCSK1N	ProSAAS	0.429507086		
IGFALS	Insulin-like growth factor-binding protein complex acid labile subunit	0.443733873		T
CLUL1	Clusterin-like protein 1	0.448261392		U
LECT2	Leukocyte cell-derived chemotaxin-2	0.458993238		
SEMA4B	Semaphorin-4B	0.463568773		U, T
RARRES1	Retinoic acid receptor responder protein 1	0.465422755		T
SEMA3A	Semaphorin-3A	0.472397218		U
SERPINA6	Corticosteroid-binding globulin	0.473269376		U
OGN	Mimecan	0.473307381		U
FSTL4	Follistatin-related protein 4	0.482381688		U
IGF2	Insulin-like growth factor II	0.486571677		
FMOD	Fibromodulin	0.492765562		

Abbreviations: U, untreated q12+ vs. q4; and T, treated q12+ vs. q4.

Supplemental Table 14. Overlapping proteins identified in Proteomics Analyses described in Supplemental Tables 11-13.

Cohort	Genes	Protein
Untreated q12+ vs. q4 + Treated q12+ vs. q4 + nnv vs.nvAMD (n=8)	HBB	Hemoglobin subunit beta
	HBA1	Hemoglobin subunit alpha
	CA1	Carbonic anhydrase 1
	ALDOA	Fructose-bisphosphate aldolase A
	KRT5	Keratin, type II cytoskeletal 5
	APOB	Apolipoprotein B-100
	HSP90AB1	Heat shock protein HSP 90-beta
	SEMA4B	Semaphorin-4B
Untreated q12+ vs. q4 + Treated q12+ vs. q4 (n=11)	FGA	Fibrinogen alpha chain
	FGG	Fibrinogen gamma chain
	NEB	Nebulin
	ADAMTS1	A disintegrin and metalloproteinase with thrombospondin motifs 1
	PEBP4	Phosphatidylethanolamine-binding protein 4
	HSPA8	Heat shock cognate 71 kDa protein
	PROZ	Vitamin K-dependent protein Z
	CD163	Scavenger receptor cysteine-rich type 1 protein M130
	CDON	Cell adhesion molecule-related/down-regulated by oncogenes
	CTSZ	Cathepsin Z
	FCGBP	IgGFc-binding protein
Treated q12+ vs. q4 + nnv vs. nvAMD (n=10)	IGFALS	Insulin-like growth factor-binding protein complex acid labile subunit
	CARTPT	Cocaine- and amphetamine-regulated transcript protein
	C4BPB	C4b-binding protein beta chain
	F11	Coagulation factor XI
	BFSP2	Phakinin
	CRYBB2	Beta-crystallin B2
	NOTCH2NLB	Notch homolog 2 N-terminal-like protein B
	C18orf63	Uncharacterized protein C18orf63
	RARRES1	Retinoic acid receptor responder protein 1
	BLVRB	Flavin reductase (NADPH)
Untreated q12+ vs. q4 + nnv vs. nvAMD (n=34)	HBD	Hemoglobin subunit delta
	PRDX2	Peroxiredoxin-2
	CA3	Carbonic anhydrase 3
	UBB	Polyubiquitin-B
	PARK7	Protein/nucleic acid deglycase DJ-1
	UCHL1	Ubiquitin carboxyl-terminal hydrolase isozyme L1
	CA2	Carbonic anhydrase 2
	PRDX1	Peroxiredoxin-1
	CFHR4	Complement factor H-related protein 4
	SERPINB6	Serpin B6
	KRT13	Keratin, type I cytoskeletal 13
	ERN1	Serine/threonine-protein kinase/endoribonuclease IRE1
	HPR	Haptoglobin-related protein
	YWHAE	14-3-3 protein epsilon
	SERPINA6	Corticosteroid-binding globulin
	OGN	Mimecan
	HEXA	Beta-hexosaminidase subunit alpha
	CRYAB	Alpha-crystallin B chain
	FABP5	Fatty acid-binding protein 5
	MIF	Macrophage migration inhibitory factor
	CRYBA4	Beta-crystallin A4
	CRYBA1	Beta-crystallin A3
	PKM	Pyruvate kinase PKM
	ANXA2P2	Putative annexin A2-like protein
	SEMA3A	Semaphorin-3A
	FCN3	Ficolin-3
	ACTG1	Actin, cytoplasmic 2
	HSPB1	Heat shock protein beta-1
	CLUL1	Clusterin-like protein 1
	VIM	Vimentin
	SORD	Sorbitol dehydrogenase
	BGN	Biglycan
	FSTL4	Follistatin-related protein 4
	S100B	Protein S100-B

Supplemental Table 15

Supplemental Table 15. Complement proteins identified Proteomics Analyses in aqueous fluid from nvAMD patients.

Complement Proteins			
**Detected in aqueous of nvAMD patients**	**Remaining after Step 1**	**Remaining after Step 2**	**Remaining after Step 3**
Complement C4-B	Complement C1q subcomponent subunit A	Complement C1q subcomponent subunit A	Complement C4-A
Complement C1q tumor necrosis factor-related protein 3	Complement C4-A	Complement C4-A	Complement factor H-related protein 4
Complement C1s subcomponent	Complement factor H-related protein 4	Complement factor H-related protein 4	Complement factor H-related protein 5
Complement component C6	Complement factor H-related protein 5	Complement factor H-related protein 5	
Complement C1r subcomponent-like protein	Complement C3	Complement C3	Complement C3
Complement C3	Complement factor H-related protein 2	Complement factor H-related protein 2	Complement factor H-related protein 2
Complement factor B	Complement factor I	Complement factor I	Complement factor I
Complement C2	Complement factor H-related protein 4	Complement factor H-related protein 4	Complement factor H-related protein 4
Complement component C8 gamma chain	Complement component C8 beta chain	Complement component C8 beta chain	Complement component C8 beta chain
Complement C1q subcomponent subunit B			
Complement factor I			
Complement component C8 alpha chain			
Complement component C7			
Complement component C8 beta chain			
Complement factor H-related protein 1			
Complement C5			
Complement factor D			
Complement C1r subcomponent			
Complement C1q subcomponent subunit C			
Complement component C9			
Complement factor H			
Complement factor H-related protein 2			
Complement C1q subcomponent subunit A			
Complement C4-A			
Complement factor H-related protein 4			
Complement factor H-related protein 5			

Untreated q4 vs. q12+ patients (blue), treated q4 vs. q12+ patients (red), nnvAMD vs. nvAMD (green).

Supplemental Table 16

Supplemental Table 16. Baseline characteristics of patients used in ApoB100 ELISA.

**Characteristic**	**Untreated nvAMD (n=10)**	**nnvAMD (n=10)**	**Non-AMD Control (n=10)**	***P***
Mean Age (yr)	81.1 ± 1.8	75.9 ± 3.0	73.5 ± 3.3	0.163
Female % (#)	50% (5)	50% (5)	40% (4)	1
Pseudophakic % (#)	70% (7)	0% (0)	30% (3)	**0.003**

Abbreviations: n, sample size; nnvAMD, non-neovascular Age-related Macular Degeneration; nvAMD, neovascular Aging Macular Degeneration; AMD, Age-related Macular Degeneration; ApoB100, apolipoprotein B100; ELISA, enzyme-linked immunoassay; and yr, years. Values displayed as mean ± standard error of the mean. Statistical analysis was performed using ANOVA and Fisher's exact test. Data in bold are statistically significant.

Supplemental Figure 1

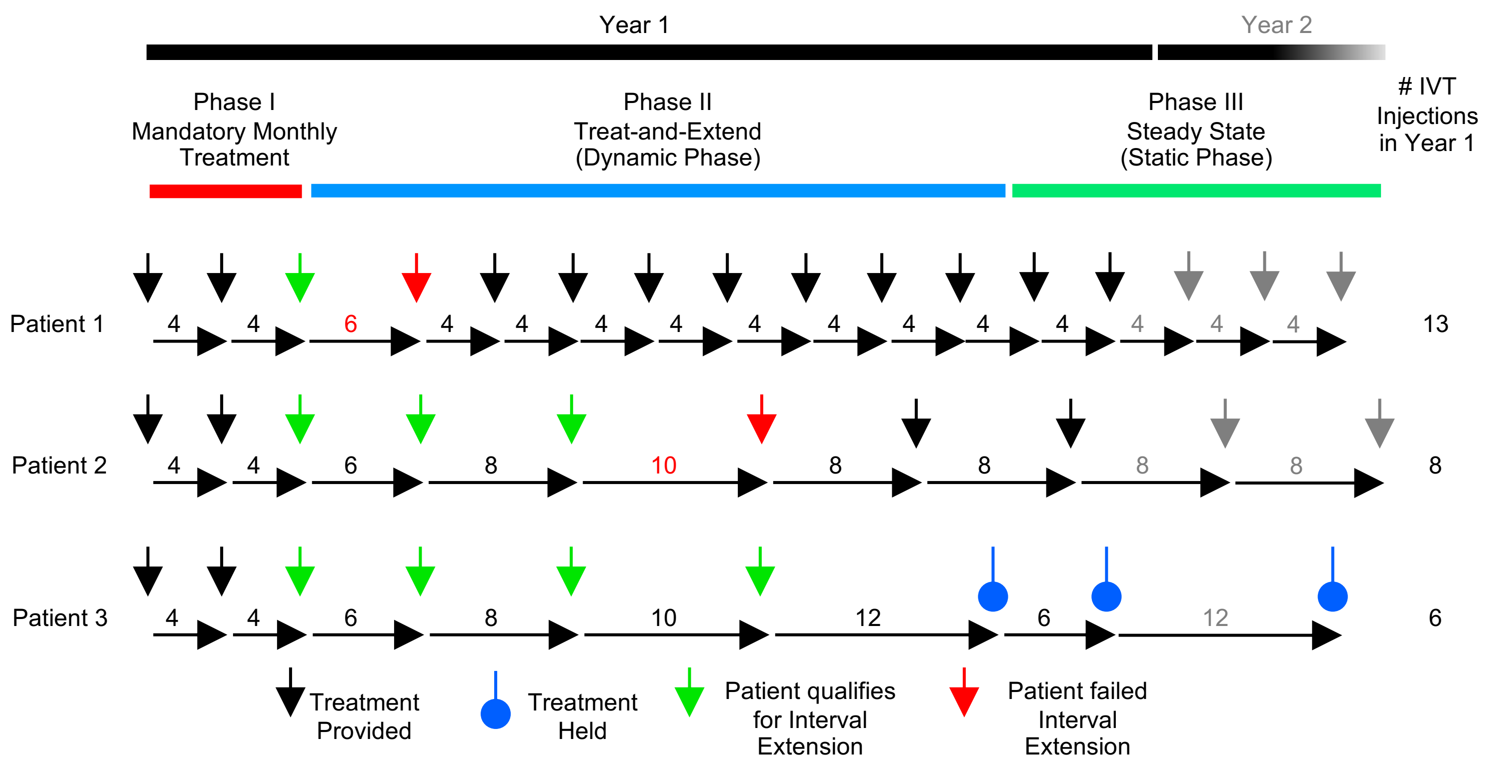

Supplemental Figure 2

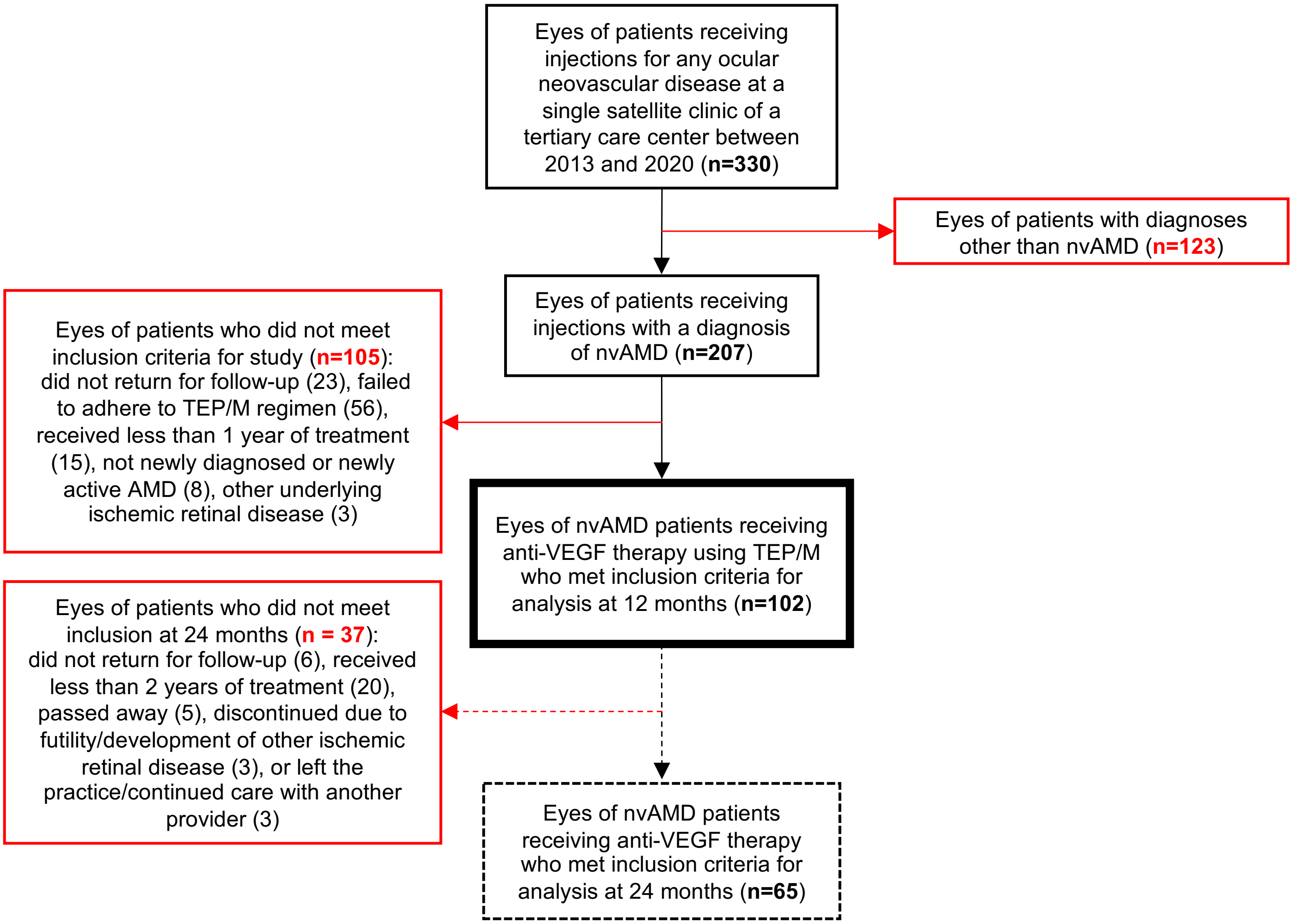

Supplemental Figure 3

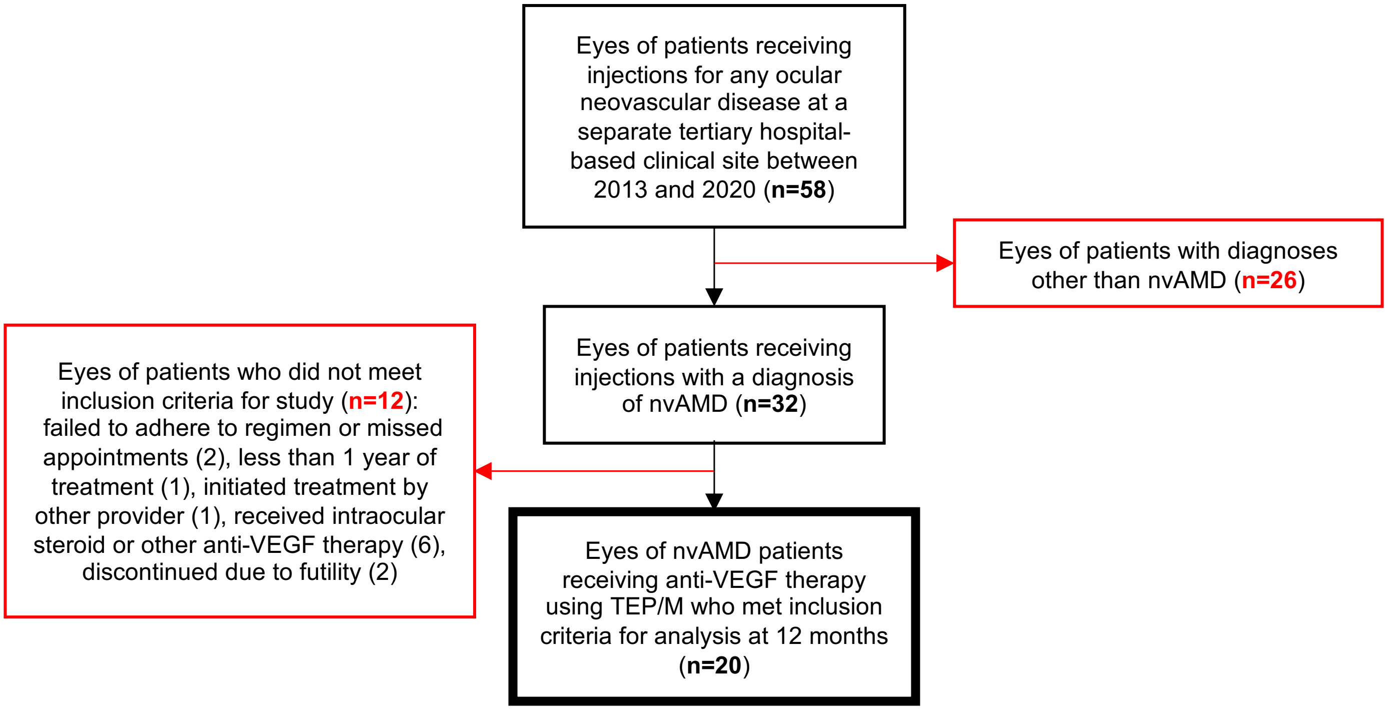

Supplemental Figure 4

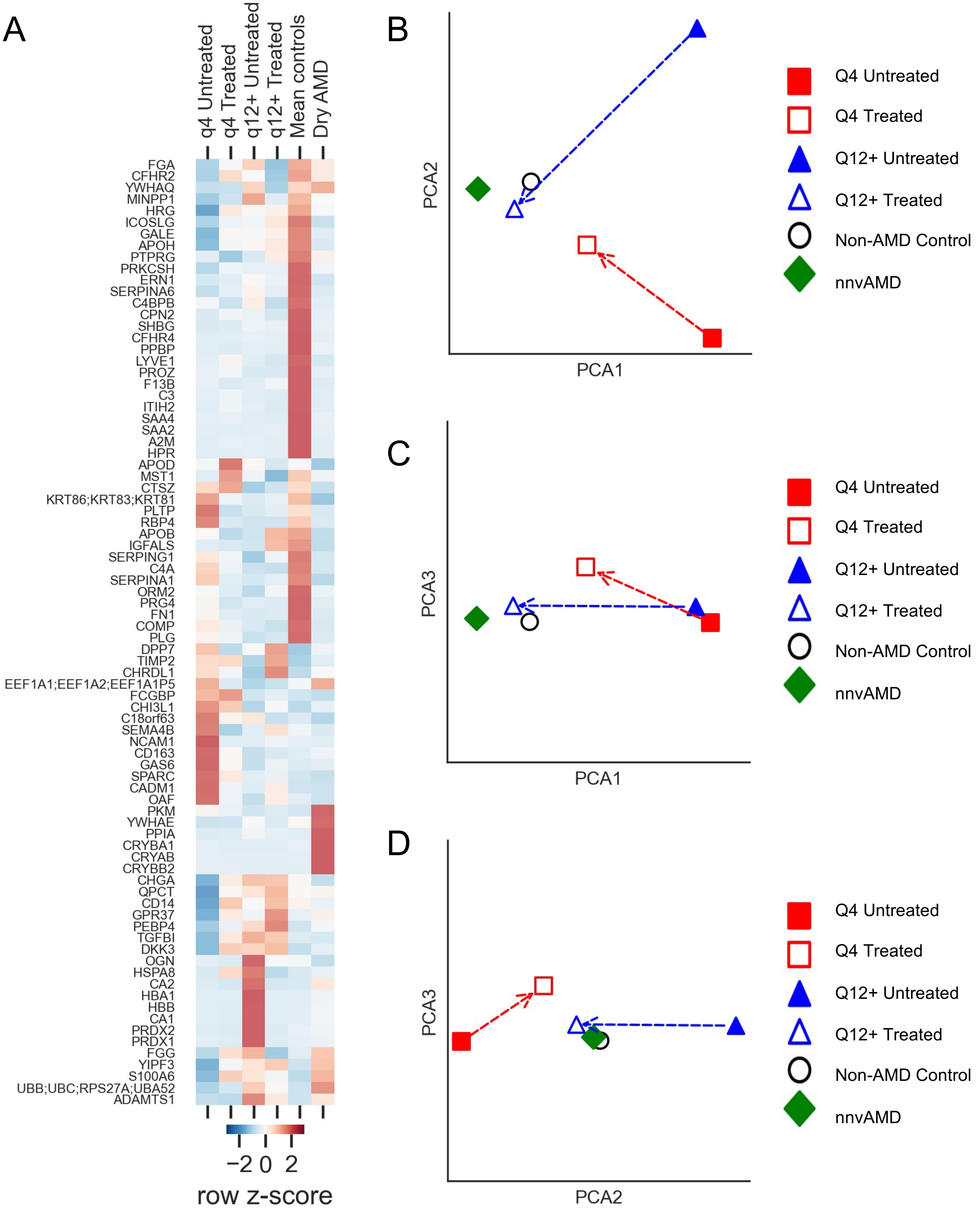

Supplemental Figure 5

A

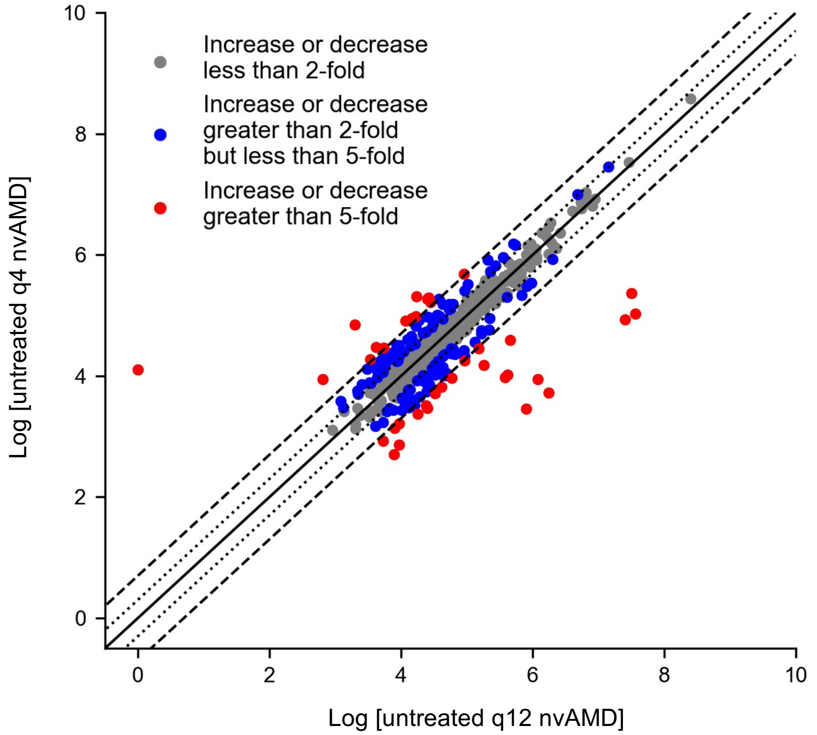

B

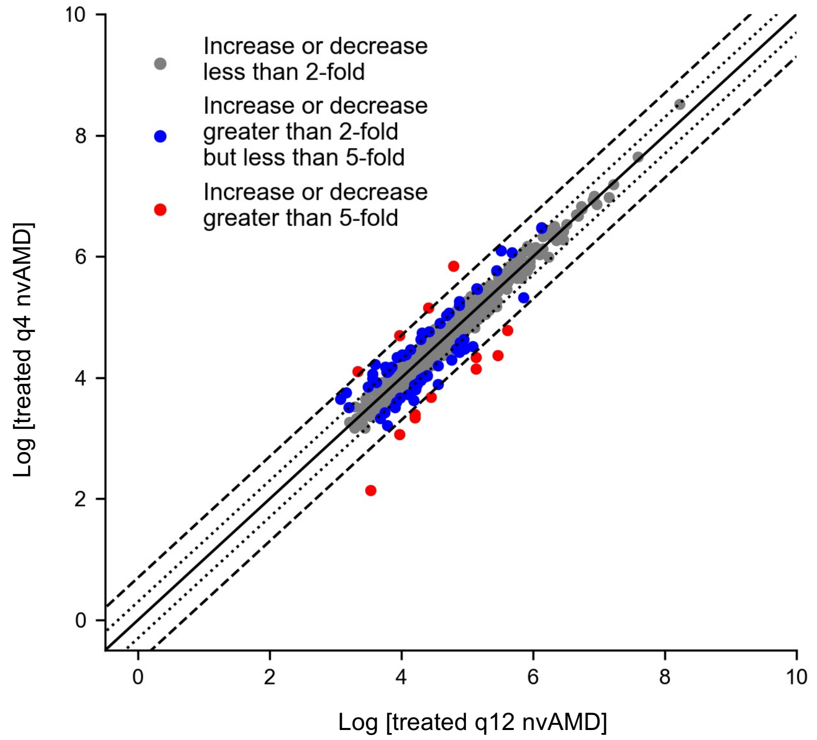

Supplemental Figure 6

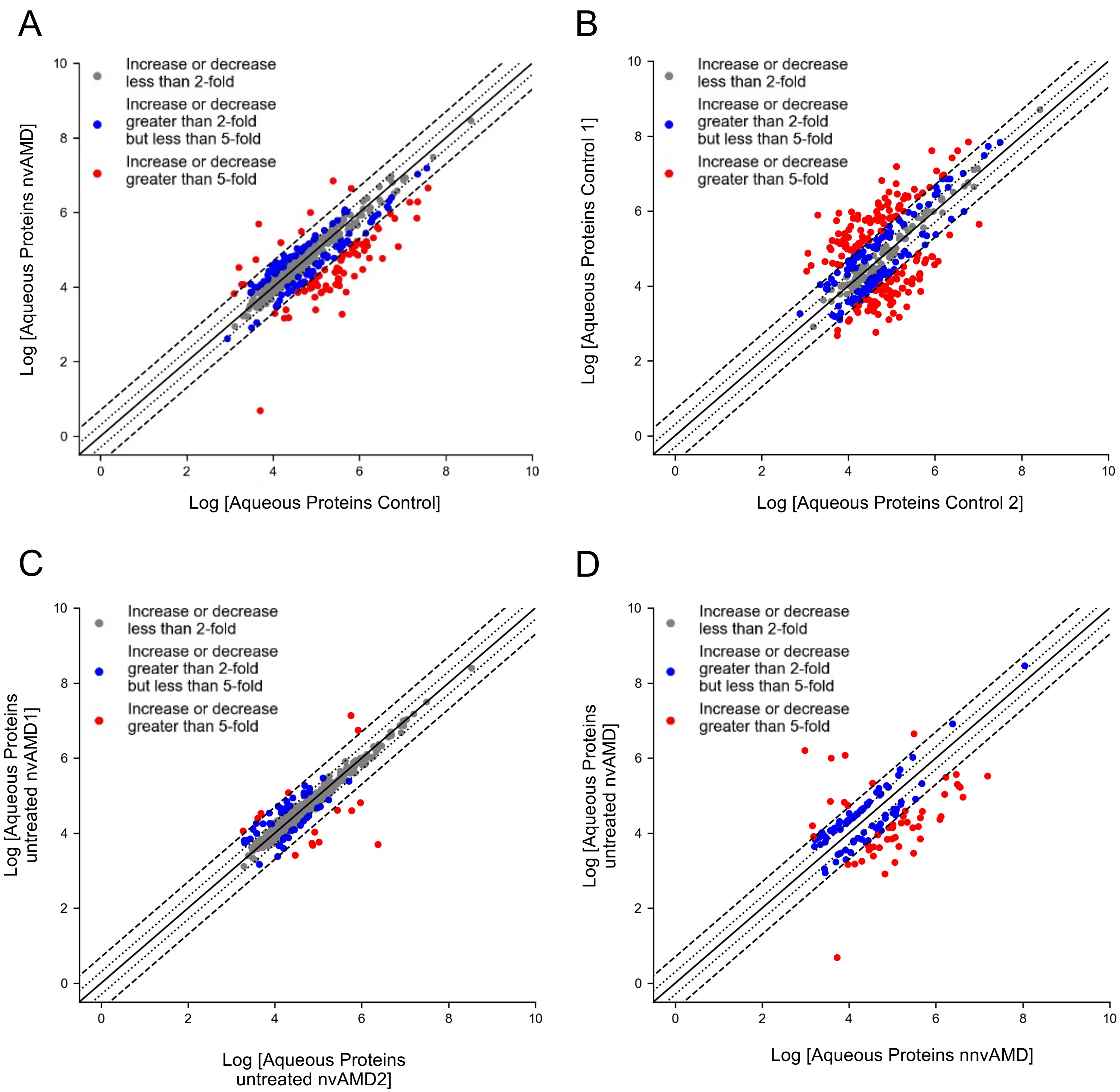

Supplemental Figure 7

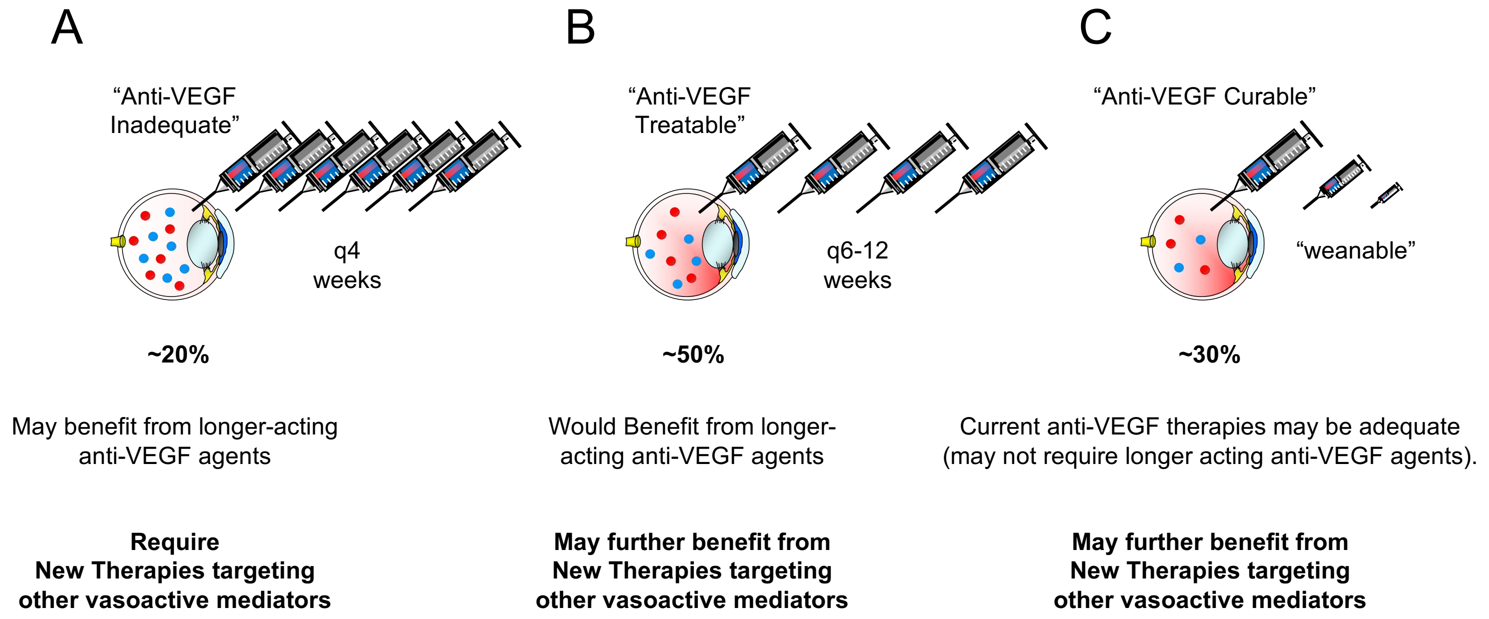

Supplement: Supplemental data [file jci-132-144469-s220.pdf]
